# Modulation of hippocampal protein expression by a brain penetrant biologic TNF-α inhibitor in the 3xTg Alzheimer’s disease mice

**DOI:** 10.1186/s12967-024-05008-x

**Published:** 2024-03-18

**Authors:** Nataraj Jagadeesan, G. Chuli Roules, Devaraj V. Chandrashekar, Joshua Yang, Sanjana Kolluru, Rachita K. Sumbria

**Affiliations:** 1 Department of Biomedical and Pharmaceutical Sciences, School of Pharmacy, Chapman University, Irvine, CA 92618 USA; 2 Rancho Cucamonga High School, 11801 Lark Dr, Rancho Cucamonga, CA 91701 USA; 3 Department of Neurology, University of California, Irvine, CA 92697 USA

**Keywords:** Biologic TNF-α inhibitor, Transferrin receptor antibody, Spatial proteomics, Alzheimer’s disease, Tau, Amyloid beta, 3xTg

## Abstract

**Background:**

Biologic TNF-α inhibitors (bTNFIs) can block cerebral TNF-α in Alzheimer’s disease (AD) if these macromolecules can cross the blood–brain barrier (BBB). Thus, a model bTNFI, the extracellular domain of type II TNF-α receptor (TNFR), which can bind to and sequester TNF-α, was fused with a mouse transferrin receptor antibody (TfRMAb) to enable brain delivery via BBB TfR-mediated transcytosis. Previously, we found TfRMAb-TNFR to be protective in a mouse model of amyloidosis (APP/PS1) and tauopathy (PS19), and herein we investigated its effects in mice that combine both amyloidosis and tauopathy (3xTg-AD).

**Methods:**

Eight-month-old female 3xTg-AD mice were injected intraperitoneally with saline (n = 11) or TfRMAb-TNFR (3 mg/kg; n = 11) three days per week for 12 weeks. Age-matched wild-type (WT) mice (n = 9) were treated similarly with saline. Brains were processed for immunostaining and high-resolution multiplex NanoString GeoMx spatial proteomics.

**Results:**

We observed regional differences in proteins relevant to Aβ, tau, and neuroinflammation in the hippocampus of 3xTg-AD mice compared with WT mice**.** From 64 target proteins studied using spatial proteomics, a comparison of the Aβ-plaque bearing vs. plaque-free regions in the 3xTg-AD mice yielded 39 differentially expressed proteins (DEP) largely related to neuroinflammation (39% of DEP) and Aβ and tau pathology combined (31% of DEP). Hippocampal spatial proteomics revealed that the majority of the proteins modulated by TfRMAb-TNFR in the 3xTg-AD mice were relevant to microglial function (⁓ 33%). TfRMAb-TNFR significantly reduced mature Aβ plaques and increased Aβ-associated microglia around larger Aβ deposits in the 3xTg-AD mice. Further, TfRMAb-TNFR increased mature Aβ plaque-associated microglial TREM2 in 3xTg-AD mice.

**Conclusion:**

Overall, despite the low visual Aβ load in the 11-month-old female 3xTg-AD mice, our results highlight region-specific AD-relevant DEP in the hippocampus of these mice. Chronic TfRMAb-TNFR dosing modulated several DEP involved in AD pathology and showed a largely microglia-centric mechanism of action in the 3xTg-AD mice.

**Supplementary Information:**

The online version contains supplementary material available at 10.1186/s12967-024-05008-x.

## Introduction

Alzheimer’s disease (AD) is the leading cause of dementia and is neuropathologically characterized by the accumulation of amyloid beta (Aβ) and hyperphosphorylated tau protein containing tau tangles [1]. Clinically, AD is characterized by progressive memory loss, personality disorder, and general cognitive decline [2]. Though the mechanisms underlying AD dementia are not entirely understood, the amyloid-cascade hypothesis is the most widely studied and places Aβ as the primary initiator of AD pathogenesis [3]. However, it is increasingly recognized that Aβ accumulation, along with other pathological processes, causes and drives AD dementia [4, 5].

In this regard, tau tangle formation follows Aβ accumulation and is more closely associated with cognitive decline in AD, and the tau hypothesis of AD suggests that tau aggregates are the primary drivers of neurodegeneration in AD [5]. Among the common processes that link Aβ and tau pathology in AD, the role of neuroinflammation has come to the fore, and the neuroinflammation hypothesis for AD posits that the inflammatory response to Aβ accumulation and tau tangles underlies neuronal damage and AD dementia [6, 7]. Additionally, inflammation may also drive Aβ accumulation and tau phosphorylation, resulting in a self-perpetuating cycle of Aβ accumulation, tau phosphorylation, and neuronal cell death [5]. Therefore, neuroinflammation appears to be a common link between Aβ and tau pathology and recent evidence shows a bi-phasic inflammatory response in the AD brain such that the first peak correlates with amyloidosis and the second peak is associated with tau pathology [8].

Among the key mediators involved in initiating and propagating neuroinflammation in AD is the pro-inflammatory cytokine, tumor necrosis factor-alpha (TNF-α), which has been strongly linked to AD progression [9, 10]. The TNF-α death domain pathway is progressively activated in the AD brain and contributes to cellular degeneration [11], and increased TNF-α in the brain is associated with the pathological features of AD [12–14]. TNF-α is colocalized with Aβ plaques in AD human brains and animal models [10, 15], and TNF-α mediated inflammation contributes to Aβ plaques and tau hyperphosphorylation [16], which result in neuronal damage and cognitive decline [17]. In addition, inhibition of soluble TNF-α signaling in AD mice prevents pre-plaque-associated neuropathology [18]. Consequently, TNF-α genetic deletion reduces plaque formation by lowering Aβ generation in AD mice [19], and genetic ablation of TNF-α receptor-1 or administration of TNF-α modulator/inhibitors to AD mice results in attenuation of the Aβ pathology [18, 20–23], suggesting that inhibition of TNF-α signaling confers a protective effect against AD pathology.

Our prior work has also shown robust protective effects of a blood–brain barrier (BBB)-penetrating biologic TNF-α inhibitor on Aβ [24, 25] and tau pathology [26]. A BBB-penetrating biologic TNF-α inhibitor was engineered to allow non-invasive transvascular delivery of this large molecule to the brain across the BBB [27, 28]. The BBB-penetrating biologic TNF-α inhibitor is a fusion protein of a monoclonal antibody against the mouse transferrin receptor (TfRMAb) and the extracellular domain of TNF-α receptor II (TNFR), a biologic TNF-α inhibitor [29]. The TfRMAb domain of the fusion protein binds to the TfR that are enriched at the BBB and enables receptor-mediated transport of the fusion protein across the BBB [29]. Accordingly, the TfRMAb-TNFR retains high-affinity binding to TfR and TNF-α [26] and readily enters the brain [29].

Given that neuroinflammation appears to be a common pathological process that can initiate and propagate Aβ accumulation and tau tangle formation, the two main prominent neuropathological features of AD, we hypothesized that the BBB-penetrating TfRMAb-TNFR will be therapeutic in a model system that combines both the Aβ and tau pathology. Therefore, after showing protective effects in Aβ [25] and phosphorylated tau-bearing mice [26], a logical next step was to elucidate the effect of the BBB-penetrable TNF-α inhibitor in the triple transgenic 3xTg-AD mice that are characterized by the presence of three Aβ and tau mutations (PS1M146V, APPSwe, and tau P301L transgenes) [30]. 3xTg-AD mice exhibit both Aβ plaques and tau tangles in an age-dependent manner within the cortex, hippocampus, and amygdala [30–34], develop neuroinflammation [35], and closely mimic human AD [30, 36], and were therefore selected for the current study.

In the current study, we treated eight-month-old 3xTg-AD female mice intraperitoneally (IP) with saline or TfRMAb-TNFR three days a week for 12 weeks. WT littermate (B6129SF/J) female mice were injected with saline. The effect of TfRMAb-TNFR on amyloidosis, phosphorylated tau, and microgliosis was studied using immunostaining. We also performed spatial hippocampal proteomics using the NanoString GeoMx Digital Spatial Profiling (DSP) technology to study the differentially regulated proteins in the 3xTg-AD mice, and the modulation of these proteins with TfRMAb-TNFR treatment.

## Materials and methods

The article follows the ARRIVE guidelines 2.0 for reporting animal research.

### Fusion protein

TfRMAb-TNFR was produced in Chinese hamster ovary cells (CHO-K1) by transient expression and purified using protein A and size-exclusion chromatography (WuXi Biologics). Protein expression was confirmed via immunoblot, as previously described [29]. Enzyme-linked immunoassays (ELISAs) were used to validate the affinity of TfRMAb-TNFR for both human TNF-α and mouse TfR [26, 29]. The TfRMAb-TNFR fusion protein was formulated in 0.01 M sodium acetate, 0.148 M NaCl, and 0.01% polysorbate 80 at pH = 5.5 to maintain stability during protein purification. The solution was sterile filtered and stored at − 80 °C until it was used.

### Mouse treatment

**Fig. 1:**
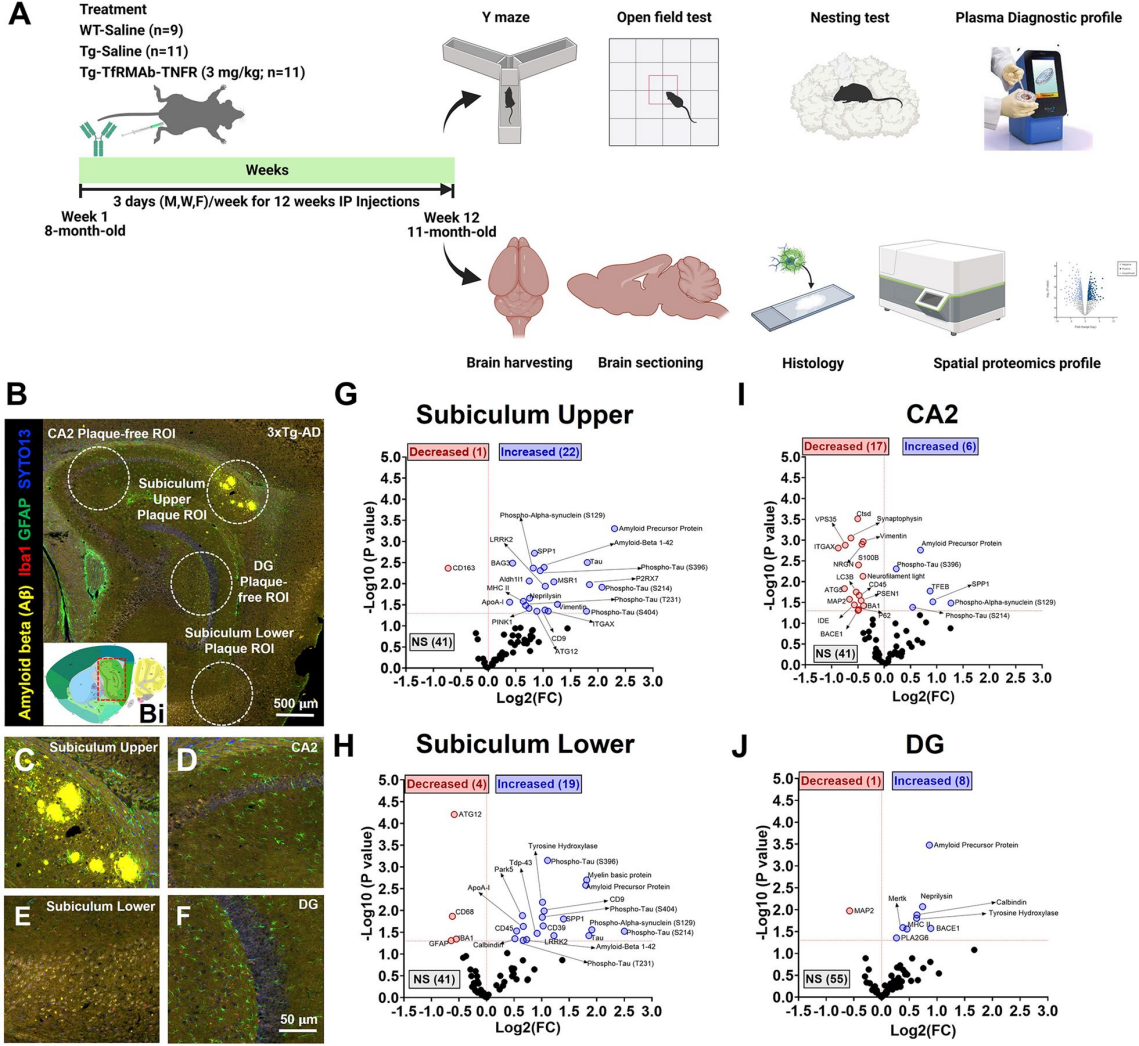
Nanostring GeoMx spatial proteomics comparing hippocampal protein expression in Tg-Saline vs. WT-Saline mice. Schematic experimental design for the study (**A**). Representative images from NanoString GeoMX DSP platform of the Tg-Saline mice showing Aβ plaque-bearing and plaque-free hippocampal regions of interest (ROIs) (the red-boxed region in the thumbnail brain section image in Bi, taken from the Allen Institute, shows the hippocampus which is shown as a high-resolution image in **B**). Scale bar = 500 μm. Subiculum upper (plaque-bearing dorsal subiculum) (**C**), CA2 (plaque-free) (**D**), subiculum lower (plaque-bearing ventral subiculum) (**E**), and DG (plaque-free) (**F**) subregions labeled with four morphology markers [Aβ (yellow), Iba1 (red), GFAP (green), and nuclear marker SYTO13 (blue)]. Scale bar = 50 μm. Volcano plots show the magnitude of change (Log2 fold) on the X-axis versus statistical significance on the Y-axis (−Log10(p value)) for 3xTg-Saline vs. WT-Saline comparisons of differentially expressed proteins. Blue dots represent upregulation, red dots represent downregulation, and black dots represent no statistical (NS) change in the subiculum upper (**G**), subiculum lower (**H**), CA2 (**I**), and DG (**J**) hippocampal regions, respectively. Data from n = 6 mice per group and 4 ROIs per mouse

The animal protocols described in this study were approved by the Chapman University Institutional Animal Care and Use Committee (IACUC, Animal Protocol # 2020-1170) and adhered to the NIH guidelines for the Care and Use of Laboratory Animals. Mice were housed and maintained in standard cages at 21 ± 2 °C with 55 ± 5% humidity with a 12:12 h light/dark cycle and given food and water ad libitum. Twenty-two eight-month-old female triple transgenic homozygous 3xTg-AD mice on a B6; 129 genetic background from Jackson Laboratory (B6;129-Tg (APPSwe, tauP301L)1Lfa *Psen1*^*tm1Mpm*^/Mmjax, Stock no. 004807, Bar Harbor), were randomly assigned to two groups and were group housed with each cage containing 4–5 mice. Randomization was done such that the average mouse weights per group did not differ significantly. 3xTg-AD mice were injected IP with either sterile saline (0.9% saline solution, Teknova) (Tg-Saline; n = 11) or TfRMAb-TNFR (Tg-TfRMAb-TNFR, 3 mg/kg; n = 11) for twelve weeks, three days a week on Mondays, Wednesdays, and Fridays. Age- and weight-matched B6129SF2/J wild-type (WT-Saline; n = 9, Stock no. 101045, Jackson Laboratory, Bar Harbor) female mice were injected with an equivalent volume of saline (Fig. 1A). Only female mice were used in the current study based on deviation of reported phenotype in male mice [35, 37–39]. After each injection, mice were examined for signs of immune response (general appearance, spontaneous locomotion, and posture), and animal body weights were recorded weekly [40]. At 11 months, behavior tests (open-field, Y-maze, and nesting) were performed over a week (Fig. 1A). Blood samples collected via the retro-orbital sinus in heparinized microhematocrit capillary tubes (Fisher Scientific) under anesthesia (Isoflurane-SomnoFlo) were centrifuged at 10,000×*g* for 5 min. The supernatant (plasma) was collected for a complete diagnostic panel using the VetScan rotor (Abaxis, #10023219) and VetScan VS2 chemical analyzer (Zoetis). Mice were injected with a lethal dose of Euthasol (150 mg/kg, IP) according to the Chapman University IACUC-approved protocol and perfused with ice-cold phosphate buffered saline (PBS). Brains were harvested and fixed in 4% paraformaldehyde (PFA) for immunostaining and spatial proteomics.

### Brain tissue preparation

Brains were removed and fixed in 4% PFA for 24 h at 4 °C, serially immersed in 10%, 20% and 30% sucrose for cryoprotection at 4 °C for 24 h each, followed by freezing and storage at − 80 °C. The frozen brains were mounted using Tissue-Tek OCT compound and cut into alternating 20 μm- and 10 μm-thick sagittal sections using a freezing cryostat (Leica freezing microtome CM3050S), and free-floating sections were stored in PBS with 0.01% sodium azide solution until assayed. The 10 μm sections were used for spatial proteomics, and the 20 μm sections were used for standard immunostaining.

### NanoString GeoMx spatial proteomic analysis

Ten-μm thick sections were mounted in the center of super frost plus slides (35.3 mm by 14.1 mm) (New Erie Scientific LLC, #260100) and allowed to dry at room temperature (RT) overnight. Slide samples were sent to NanoString (Seattle) at ambient temperature for high-resolution multiplexing using the GeoMx DSP. For this, slide-mounted mouse brain sections prepared above were incubated with a cocktail of antibodies conjugated to UV-photocleavable oligo tags. A total of 64 target proteins included in the NanoString AD pathology, AD pathology extended, Parkinson’s disease (PD), autophagy, glial cell typing, and neural cell typing modules were studied. Sections were also stained with antibodies against Aβ42 (MOAB2, Novus USA), microglia (Iba1, Clone 20A12.1, Millipore Sigma), and astrocytes [glial fibrillary acidic protein (GFAP, Clone GA5, Novus USA)], along with the nucleic acid binding fluorescent dye, SYTO 13, as morphological markers for delineation of regions of interest (ROIs). Circular Aβ plaque-bearing and plaque-free ROIs (600 µm in diameter) were selected in the hippocampus, with the CA2 and dentate gyrus (DG) designated as the plaque-free ROIs, and the subiculum upper and subiculum lower designated as the Aβ plaque-bearing ROIs, based on the Aβ plaque localization in these mice (Fig. 1B). These selected areas were illuminated individually via UV light to cleave the UV-photocleavable oligo tags conjugated to the antibodies resulting in spatially mapped counts that were read by the nCounter® platform [41, 42]. Digital counts were first normalized with isotype control antibodies and further normalized to WT-Saline protein count in the specific ROI area. Normalized counts were then compared between the WT-Saline and Tg-Saline mice, Tg-Saline and Tg-TfRMAb-TNFR mice, or between the plaque-bearing and plaque-free ROIs in the Tg-Saline mice.

### Aβ, Iba1, and TREM2 immunofluorescence

Three free-floating brain sections of 20-µm thickness and 600 µm apart were washed with PBS three times for 5 min each, incubated in 70% formic acid for 10 min at RT, washed with deionized water and blocked with 0.5% bovine serum albumin (BSA) in PBS containing 0.3% TritonX-100 for 1 h at RT. Sections were then incubated in Alexa Fluor 488-conjugated 6E10 (1:1000, BioLegend, #SIG-39347) and Iba1 (1:1000, Wako, #019-19741) antibodies in 0.5% BSA with 0.3% TritonX-100 in PBS at 4 °C overnight to stain Aβ and microglia, respectively. Iba1 was selected as the microglial marker because microglial activation is associated with an increase in Iba1 [43]. Sections were washed with PBS and incubated in the dark with Alexa Fluor® 647 donkey anti-rabbit IgG (H+L) highly cross-adsorbed secondary antibody (1:500, Invitrogen, #A31573) in PBS containing 0.5% BSA with 0.3% TritonX-100 for 1 h at RT for Iba1 detection. Sections were washed with distilled water, coverslipped with Vectamount aqueous mounting media (Vector Laboratories), sealed with nail polish, and stored at 4 °C until imaging. Stained brain tissue sections were imaged using a BZ-X710 Keyence Microscope (Keyence) under a 4X objective to capture the entire hippocampus for the 6E10 quantification. For this, images were quantified by NIH Image J software (Version 1.53) for the number of positive stains/µm^2^ of the brain and stain-positive area expressed as a percentage of the total analyzed area. All images were analyzed by two observers blinded to the experimental group.

The triggering receptor expressed on myeloid cells-2 (TREM2) is an immune receptor found in microglia. For TREM2 and Iba1 dual staining, 20 μm brain sections were washed with PBS and incubated in sodium citrate buffer at 90 °C for 15 min for antigen retrieval. The sections were washed with deionized water and subjected to permeabilization with 0.3% Triton X-100 in PBS for 60 min at RT and blocked with 0.5% BSA with 0.3% TritonX-100 in PBS for 2 h. The sections were then incubated with anti-Iba1 rabbit antibody (1:1000 Wako, #019-19741) and anti-TREM2 sheep antibody (1:250, R&D system, #AF1729) in 0.5% BSA with 0.3% TritonX-100 in PBS overnight at 4 °C with gentle shaking. After washing in PBS, brain sections were incubated in anti-rabbit Alexa Fluor 488 for Iba1 (1:500, BioLegend, #40641) or anti-sheep Alexa Fluor 647 for TREM2 (1:500, Thermo Scientific, #A21448) for 2 h at RT with gentle shaking. The sections were washed with distilled water, mounted on slides, coverslipped using Vectamount aqueous mounting media (Vector Laboratories), and sealed with nail polish. Slides were stored in the dark at 4 °C until imaging.

### 6E10 and Iba1 quantification

6E10 and Iba1 co-stained sections were imaged using a Nikon Ti-E Confocal Microscope (Nikon Instruments Inc) with NIS element software. Laser and detector settings were maintained constant for the acquisition of each image. Images were captured under a 40X oil immersion objective at 1024 × 1024 pixels. For 6E10 and Iba1 dual immunostaining, two regions in the dorsal subiculum from three brain sections per mouse were imaged and analyzed using NIH ImageJ. A blinded observer determined the Iba1- and 6E10-positive areas in each image and then selected five Aβ stains per mouse in each image [44]. Each 6E10-positive Aβ stain was manually outlined to include the dense core of the 6E10-positive Aβ stain and the associated microglia [24]. 6E10-associated Iba1 mean fluorescent intensity (MFI) and area for each stain was determined using NIH ImageJ as a measure of microglial activation given the expected increase in Iba1 immunoreactivity with microglial activation [43, 45]. 6E10-positive Aβ stains were then categorized based on size into < 25 μm^2^, 25–500 μm^2^, and > 500 μm^2^ to determine the association between 6E10-associated microglia MFI and 6E10-stain size. 6E10-associated Iba1 area normalized to 6E10 area was also calculated as a measure of microglial association with Aβ. Similarly, intraneuronal 6E10-positive stains were manually outlined to quantify the intraneuronal 6E10-positive area.

### Iba1, TREM2, and Aβ quantification

TREM2 and Iba1 co-stained sections were imaged using a Nikon Ti-E Confocal Microscope (Nikon Instruments Inc) with NIS element software. Laser and detector settings were maintained constant for the acquisition of each image. Images were captured under a 40X oil immersion objective at 1024 × 1024 pixels. Sections stained for TREM2 and Iba1 were further illuminated with the 405 nm laser to detect mature Aβ plaques by the autofluorescence generated by the β-sheet-rich structures [46]. Two distinct regions in each hippocampus, one being plaque-bearing and the other being plaque-free, from three mouse brain sections per mouse were imaged and analyzed using NIH ImageJ using a threshold setting to calculate tissue area positive for TREM2, Iba1, and Aβ plaques. A blinded observer selected five Aβ plaques per mouse in each image. Circular regions of interest encompassing each Aβ plaque and the associated microglia were drawn to include microglial soma and processes associated with the plaque [24]. Aβ plaque-associated Iba1 MFI and Aβ plaque-associated TREM2 MFI for each plaque was determined using NIH ImageJ.

### Statistical analysis

GraphPad Prism (Version 9, GraphPad Software) was used for generating graphs of data and statistical analysis. The results are expressed as mean ± SEM, and outliers were identified and excluded based on the Grubb’s test, and a complete list of outliers removed is shown in Additional file 1: Table S1. For sample sizes less than 30, normality was confirmed by the D’Agostino & Pearson test. Non-parametric tests were used for non-normal data. For sample sizes greater than 30, parametric tests were used based on the assumptions of the Central Limit Theorem [47]. Two independent groups with numerical data were compared using the unpaired t-test or the Mann–Whitney U test. For comparisons between more than two independent groups with numerical data, one-way analysis of variance (ANOVA) followed by Holm Sidak’s post-hoc test [48] or Kruskal–Wallis followed by Dunn’s post-hoc test were used. For matched numerical data with two independent variables, two-way repeated measures ANOVA followed by Holm Sidak’s post-hoc test, was used. Pearson’s correlation coefficient was used to determine the correlation between two numerical variables, and Fisher’s exact test was used to compare categorical variables between two groups. Normalized NanoString counts were represented as log2fold change vs. statistical significance [−Log10 (p value)]. G*power was used for sample size estimation before the study to compare two independent groups (Tg-Saline and Tg-TfRMAb-TNFR) using a power of 80%, standard deviation of ⁓ 20–30%, significance level of 0.05, and difference in mean of 35–40%, which resulted in a sample size of 6–13 mice per group. A p ≤ 0.05 was considered statistically significant.

## Results

### AD-relevant differentially expressed proteins (DEP) were observed in the hippocampus of 3xTg-AD mice using NanoString GeoMx DSP proteomics

We performed spatial proteomics to study the DEP in the hippocampus of Tg-Saline and WT-Saline mice in the subiculum, CA2, and DG ROIs. We used the NanoString GeoMx DSP platform to get a detailed neural cell profile that allowed for spatial analysis of the expression of 64 different proteins with nCounter digital quantification. We analyzed 600 µm circular ROIs around Aβ plaques (plaque-bearing ROIs defined as the upper and lower subiculum ROIs) and the surrounding microenvironment (plaque-free ROIs defined as the DG and CA2 ROIs) (Fig. 1B–F, Additional file 2: Fig. S1). In the upper subiculum (plaque-bearing) ROI, we identified 23 DEP between the Tg-Saline and WT-Saline groups (Fig. 1G, Additional file 3: Fig. S2), out of which 1 was downregulated and 22 were upregulated. Out of these DEP, 13% were relevant to Aβ processing and degradation, 22% were relevant to tau pathology, 39% were relevant to neuroinflammation, and 26% were relevant to autophagy and PD-related proteins. In the lower subiculum (plaque-bearing) ROI, 23 DEP were identified, 4 downregulated and 19 upregulated, in the Tg-Saline mice compared to WT-Saline controls. Out of these DEP, 17% were relevant to Aβ processing and degradation, 22% were relevant to tau pathology, 30% were relevant to neuroinflammation, and 30% were relevant to autophagy, neurodegeneration, and PD-related proteins combined (Fig. 1H, Additional file 4: Fig. S3). Among the plaque-free ROIs, 23 DEP were identified in the CA2 ROI out of which 17 were downregulated, and 6 were upregulated in the Tg-Saline mice compared to the WT-Saline mice. Out of these DEP, 22% were relevant to Aβ processing and degradation, 9% were relevant to tau pathology, 30% were relevant to neuroinflammation, and 39% were relevant to autophagy, neurodegeneration, and PD-related proteins combined (Fig. 1I, Additional file 5: Fig. S4). Lastly, we identified 9 DEP in the DG (plaque-free) ROI out of which, 1 was downregulated and 8 were upregulated in the Tg-Saline mice compared with WT-Saline mice. Out of these DEP, 44% were relevant to Aβ processing and degradation, 22% were relevant to neuroinflammation, and 33% were relevant to neurodegeneration and PD-related proteins combined (Fig. 1J, Additional file 6: Fig. S5).

### Plaque-bearing versus plaque-free hippocampal regions show differential protein expression in the 3xTg-AD mice using NanoString GeoMx DSP proteomics

**Fig. 2:**
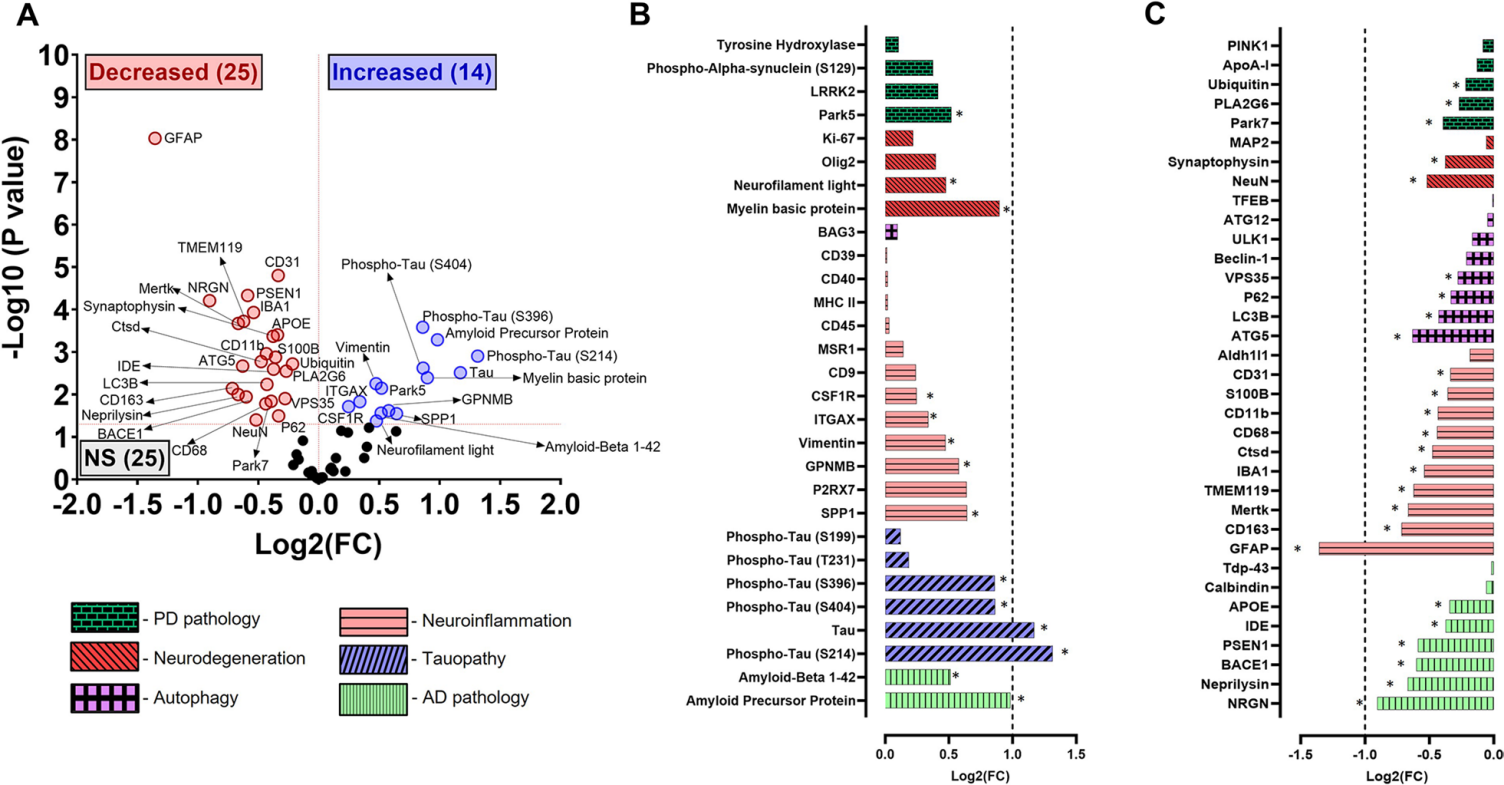
Nanostring GeoMx spatial proteomics comparing hippocampal protein expression in Aβ plaque-bearing vs. plaque-free regions of the 3xTg-AD mice. Volcano plots show the magnitude of change (Log2fold) on the X-axis versus statistical significance on the Y-axis (-Log10(P value)) for plaque-bearing vs. plaque-free comparisons of differentially expressed proteins in 3xTg-AD mice (**A**). Blue dots represent upregulation, red dots represent downregulation, and black dots represent no statistical (NS) change. Bar plots show log2 fold-change (FC) in protein expression: upregulation (**B**) and downregulation (**C**) in the hippocampus. *p < 0.05. Data from n = 6 mice per group and 4 ROIs per mouse

To determine protein changes in the Aβ plaque-bearing versus plaque-free regions of 3xTg-AD mice, we compared the DEP in the subiculum with the DG and CA2 regions; the former being the plaque-bearing region and the latter being the plaque-free regions. Our data revealed a differential pattern of protein expression between the plaque-bearing and plaque-free regions (Fig. 2). Volcano plots show 39 DEP, with 14 upregulated and 25 downregulated proteins (Fig. 2A). Out of these, 12 proteins (31%) were related to hallmark AD pathology (Aβ and tau), including 6 upregulated proteins (APP, Aβ1-42, pTauS214, total tau, pTauS404, and pTauS396) (Fig. 2B) and 6 downregulated proteins (neurogranin (NRGN), neprilysin, BACE1, PSEN1, IDE, APOE) (Fig. 2C) in the plaque-bearing versus plaque-free regions. Out of 15 (38%) neuroinflammation-related proteins, 5 were upregulated (SPP1, GPNMB, Vimentin, ITGAX, and CSF1R) (Fig. 2B), and 10 were downregulated (GFAP, CD163, Mertk, TMEM119, Iba1, Ctsd, CD68, CD11b, S100B and CD31) (Fig. 2C) in the plaque-bearing versus plaque-free regions. Out of the lysosomal autophagy proteins, 4 (10%) were downregulated in plaque-bearing versus plaque-free regions, including Atg5, LC3B, P62, and VPS35 (Fig. 2C). Out of 4 (10%) neurodegeneration-related proteins, 2 were upregulated (myelin basic protein (MBP) and neurofilament light) (Fig. 2B), and 2 were downregulated (NeuN and synaptophysin) (Fig. 2C) in plaque-bearing versus plaque-free regions. Out of 4 (10%) PD pathology-related proteins, Park5 was upregulated (Fig. 2B), whereas 3 proteins were downregulated (Park7, PLA2G6, and Ubiquitin) (Fig. 2C) in plaque-bearing versus plaque-free regions.

### TfRMAb-TNFR modulates AD-relevant pathways in the hippocampus of 3xTg-AD mice

**Fig. 3:**
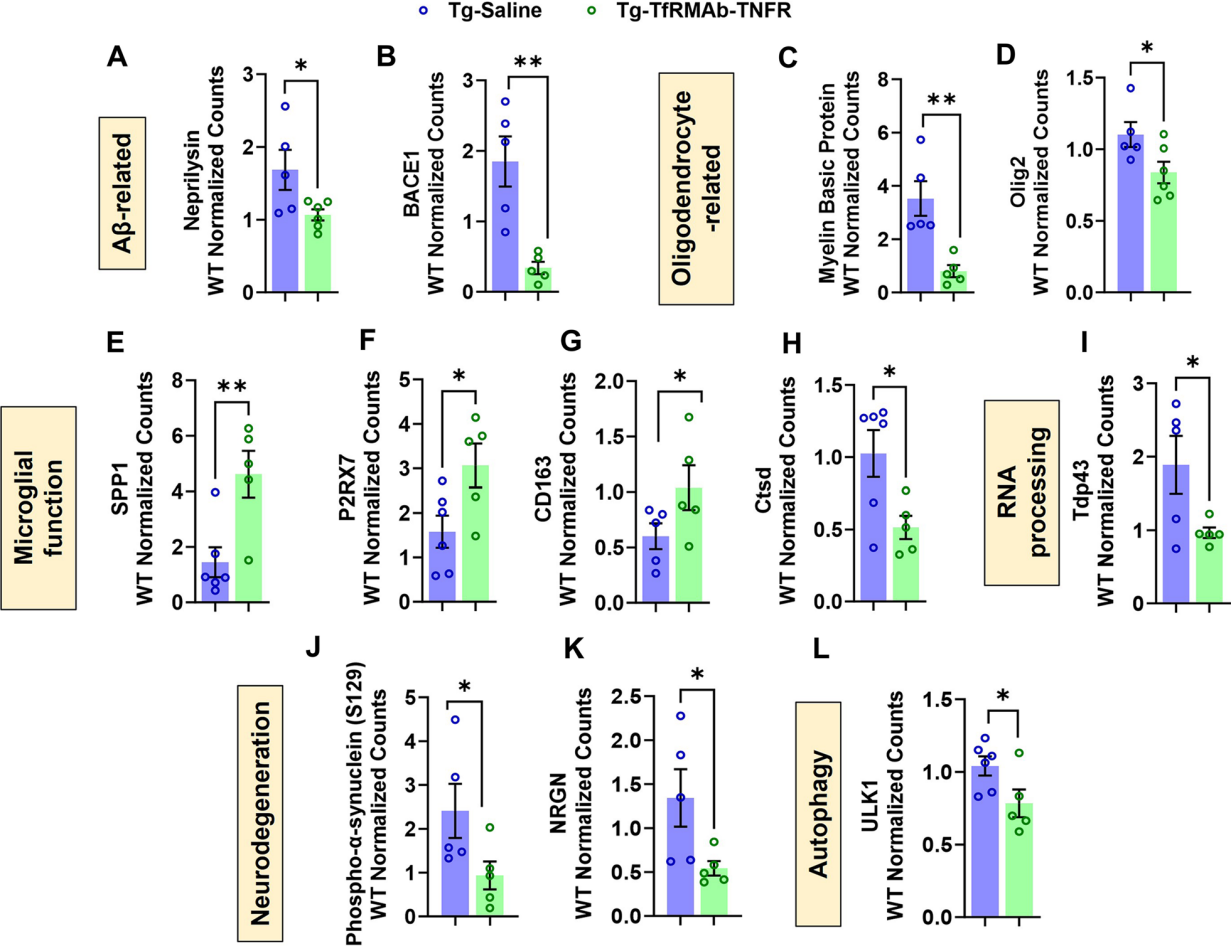
Nanostring GeoMx spatial proteomics comparing hippocampal protein expression in Tg-TfRMAb-TNFR and Tg-Saline 3xTg-AD mice. Differentially regulated proteins related to: Aβ [neprilysin (A) and β-secretase 1 (BACE1) (**B**)], oligodendrocytes [myelin basic protein (**C**) and oligodendrocyte transcription factor 2 (Oligo2, **D**)], microglial-function (secreted phosphoprotein 1 (SPP1, **E**), P2x purinoceptor 7 (P2RX7, **F**), cluster of differentiation 163 (CD163, **G**), cathepsin D (Ctsd, **H**)), RNA processing (TAR DNA-binding protein 43 (Tdp43, **I**)), neurodegeneration (phospho-α-synuclein (S129, **J**), neurogranin (NRGN, **K**)), and autophagy (Unc-51-like kinase 1 (ULK1, **L**)). Protein expression was studied in the subiculum (upper and lower), CA2, and DG. The data are shown as Mean ± SEM of n = 5–6 mice per group and were analyzed using the Mann–Whitney U test. Outliers have been detailed in Additional file 1: Table S1. *p < 0.05 and **p < 0.01 for the indicated comparisons

Next, we sought to delineate the different pathways modulated by TfRMAb-TNFR in the different regions (subiculum upper and lower, CA2, and DG) of the hippocampus of 3xTg-AD mice. TfRMAb-TNFR treatment decreased the levels of neprilysin (Fig. 3A) and β-secretase (BACE1) (Fig. 3B), proteins involved in amyloid degradation and Aβ processing, respectively, in the 3xTg-AD mice compared with saline treatment. With respect to the proteins involved in oligodendrocyte differentiation and remyelination, we found that the expression of MBP (Fig. 3C) and Olig2 (Fig. 3D) was downregulated in Tg-TfRMAb-TNFR mice compared with the Tg-Saline mice. Furthermore, microglial-associated proteins, SPP1 (Fig. 3E), P2RX7 (Fig. 3F), CD163 (Fig. 3G) were increased and Ctsd was decreased (Fig. 3H) with TfRMAb-TNFR treatment. TfRMAb-TNFR decreased the protein expression of Tdp-43 (Fig. 3I), which is associated with increased pathological tau [49]. With respect to proteins involved in neurodegeneration, we found decreased phospho-α-syn (S129) (Fig. 3J) and NRGN (Fig. 3K) in the Tg-TfRMAb-TNFR mice compared with Tg-Saline mice. In addition, the autophagy marker, ULK1, was significantly lower in Tg-TfRMAb-TNFR mice compared with Tg-Saline mice (Fig. 3L). Overall, these results showed that Tg-TfRMAb-TNFR treatment modulates several AD-relevant signaling pathways related to Aβ processing and degradation, oligodendrocytes, microglia, RNA processing, neuronal loss, and autophagy, with majority of the proteins being relevant to microglial function.

### Chronic TfRMAb-TNFR alters microglial association to Aβ deposits in 3xTg-AD mice

**Fig. 4:**
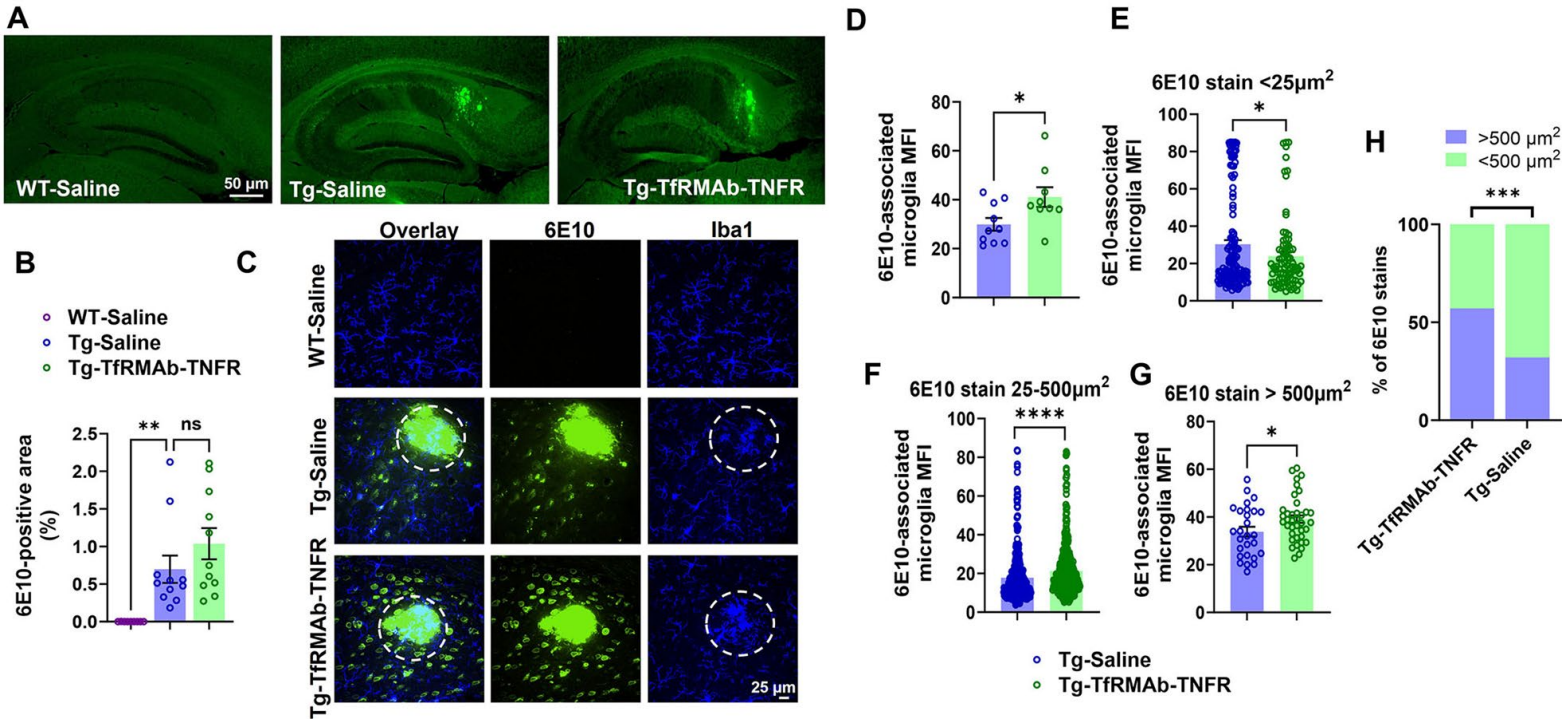
Effect of TfRMAb-TNFR on 6E10-positive Aβ load and Aβ-microglial co-localization in the hippocampus of 3xTg-AD mice. Representative images of 6E10-positive Aβ stain (**A**) and the corresponding 6E10-positive area in the entire hippocampus (**B**). Representative confocal images of 6E10 (green) and Iba1 (blue) double immunofluorescence staining in the plaque-bearing subiculum of WT-Saline and 3xTg-AD mice with or without TfRMAb-TNFR treatment (**C**). 6E10-labeled Aβ-associated microglia mean fluorescent intensity (MFI) for all 6E10 stain sizes averaged per mouse (**D**), for individual 6E10 stains < 25 μm^2^ (**E**), for individual 6E10 stains between 25–500 μm^2^ (**F**), and for individual 6E10 stains > 500 μm^2^ (**G**). The number of small (< 500 µm^2^) and large (> 500 µm^2^) 6E10 stains in the hippocampus of 3xTg-AD mice with or without TfRMAb-TNFR treatment (**H**). For **E**–**H**, 6E10 stains from all the mice per group were pooled together. Scale bars = 25–50 μm as indicated. The data are shown as Mean ± SEM for WT-Saline (n = 9), Tg-Saline (n = 11), and Tg-TfRMAb-TNFR (n = 11) mice in B-G and as bar graphs in H. Data were analyzed using the Kruskal–Wallis with Dunn’s post-hoc test or unpaired t-test in **B**–**G** compared to Tg-Saline mice, and using the Fisher’s exact test in **H**. Outliers have been detailed in Additional file 1: Table. S1. *p < 0.05, ***p < 0.001, ****p < 0.0001, and ns = not significant for the indicated comparisons

To further explore the effect of TfRMAb-TNFR on microglia- and Aβ-related pathways, Aβ staining was performed using the 6E10 antibody, which stains all forms of Aβ including the precursor form (APP). In the current study, Aβ deposits were observed primarily in the subiculum, consistent with previous work [50], with limited Aβ deposits in the cortical region (data not shown). Our results showed no significant difference in the 6E10-positive Aβ area in the Tg-TfRMAb-TNFR mice compared with Tg-Saline mice (Fig. 4A, B). Similarly, the number of total 6E10-positive stains or intraneuronal 6E10-positive area was not significantly altered by TfRMAb-TNFR treatment (Additional file 7: Fig. S6). However, we found increased 6E10-associated microglia in the Tg-TfRMAb-TNFR mice compared to the Tg-Saline mice (Fig. 4C, D; p < 0.05). To explore this association more, all the individual 6E10-positive stains per experimental group were pooled and distributed by size. We observed that the 6E10-associated microglial MFI was significantly (p < 0.05) lower for stains < 25 µm^2^ (Fig. 4E) but was significantly (p < 0.05) higher for stains > 25 µm^2^ (Fig. 4F, G) with TfRMAb-TNFR treatment. Further, the number of smaller (< 500 µm^2^) 6E10-positive stains was significantly lower while the number of larger (> 500 µm^2^) 6E10-positive stains was significantly higher with TfRMAb-TNFR treatment (Fig. 4H). Apart from Aβ, we used the AT8 antibody, specific for tau phosphorylated at ser202/thr205, to determine the impact of the treatment on tau phosphorylation. AT8-positive neurons detected in the subiculum of Tg-Saline 3xTg-AD mice and Tg-TfRMAb-TNFR 3xTg-AD mice are shown in Additional File 8: Fig. S7A, and negligible AT8-positive neurons were found in the WT-Saline mice, as expected. There were no significant changes in the AT8-positive area in Tg-TfRMAb-TNFR mice compared to Tg-Saline mice (Additional file 8: Fig. S7B).

### Chronic TfRMAb-TNFR treatment increases TREM2-positive microglial association with β-sheet-rich Aβ deposits

**Fig. 5:**
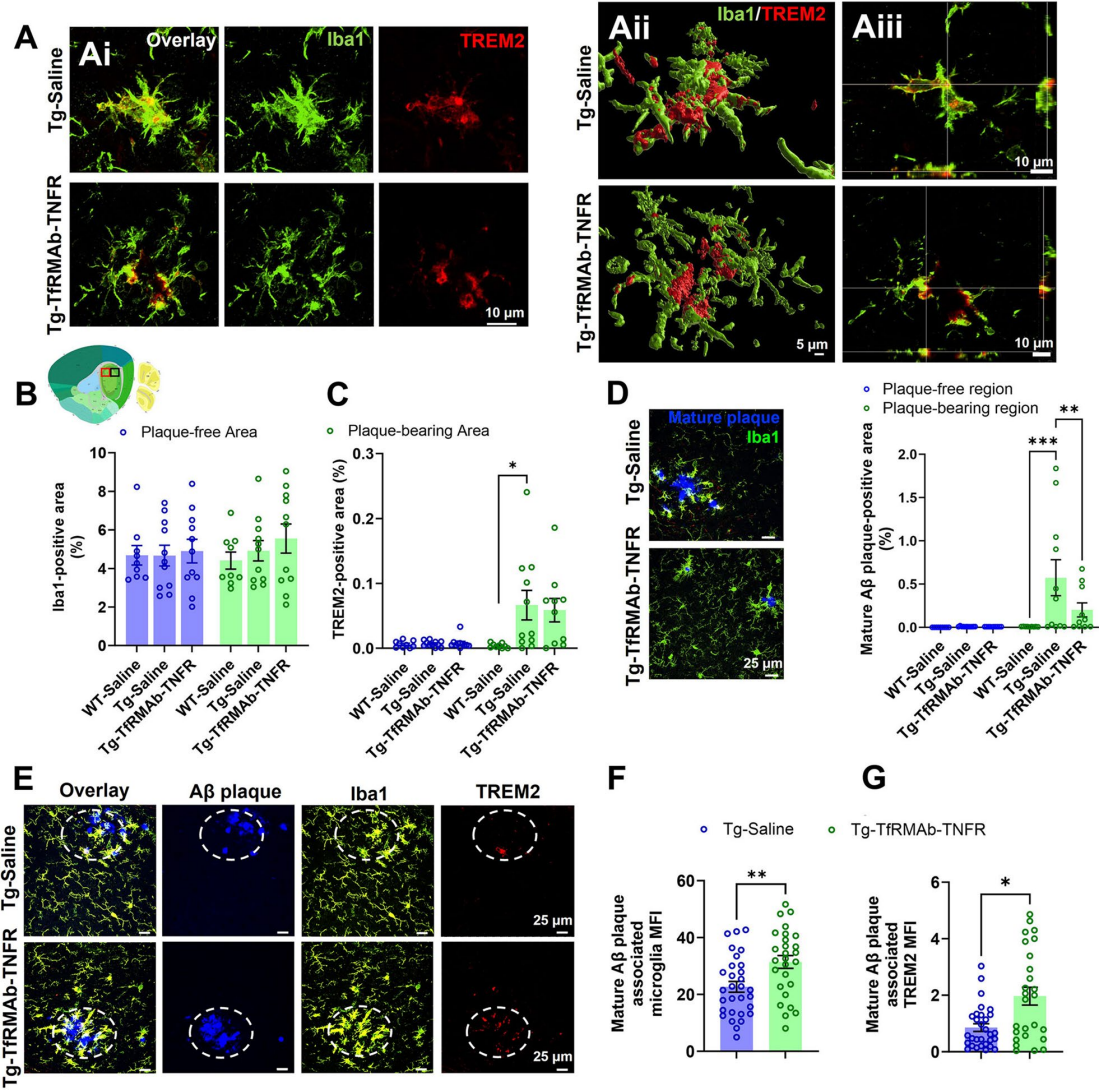
Iba1, TREM2, and β-sheet rich Aβ plaque in the hippocampus of 3xTg-AD mice with TfRMAb-TNFR treatment. Representative confocal images of Iba1-positive microglia (green) and TREM2-positive microglia (red) of the 3xTg-AD mice with or without TfRMAb-TNFR-treatment (**Ai**). Representative 3D rendering of z-stack images generated using Imaris software (**Aii**) and an orthogonal view (**Aiii**) showing Iba1 (green) and TREM2 (red) colocalization. Scale bar = 5–10 µm in A as indicated. Plaque-bearing (subiculum) black-boxed region and plaque-free (CA2) red-boxed region in the thumbnail brain section image in B were imaged to quantify Iba1-positive area % (**B**) and TREM2-positive area % (**C**). The thumbnail image in B was taken from the Allen Institute. Representative images of β-sheet-rich Aβ plaques (blue) and microglia (Iba1, green) staining in the plaque-bearing subiculum of Tg-Saline or Tg-TfRMAb-TNFR mice and the resulting β-sheet-rich Aβ plaque-positive area % (**D**). Representative confocal images showing Iba1 (green), TREM2 (red), and β-sheet-rich Aβ (blue) fluorescence staining in the plaque-bearing subiculum of 3xTg-AD mice with or without TfRMAb-TNFR treatment (**E**). Scale bar = 25 μm in **D**–**E**. Mature Aβ plaque-associated microglial MFI (**F**) and mature Aβ plaque-associated TREM2 MFI (**G**). Quantifications in F and G are based on the circular regions of interest outlined in **E**. The data are shown as Mean ± SEM for WT-Saline (n = 9), Tg-Saline (n = 11), and Tg-TfRMAb-TNFR (n = 11) mice. Data were analyzed using the two-way repeated measures ANOVA with Holm Sidak’s post-hoc test in **B**–**D**, and unpaired t-test and Mann–Whitney U test in **F**, **G**, respectively. Outliers have been detailed in Additional file 1: Table. S1. *p < 0.05, **p < 0.01, and ***p < 0.001 for the indicated comparisons

Next, we examined the association between microglia, TREM2, and mature β-sheet rich Aβ deposits. Representative confocal images with microglia and TREM2 co-localization and high-resolution 3D reconstructions of the confocal z-stacks and orthogonal images to show Iba1 and TREM2 co-localization in Tg-Saline and Tg-TfRMAb-TNFR 3xTg-AD mice are shown in Fig. 5A. Quantification of the Iba1-positive area showed no change between WT-Saline and Tg-Saline mice or between Tg-Saline mice and Tg-TfRMAb-TNFR mice in the plaque-free or plaque-bearing regions of the hippocampus (Fig. 5B). TREM2-positive area also remained unchanged between WT-Saline and Tg-Saline mice or between Tg-Saline mice and Tg-TfRMAb-TNFR mice in the plaque-free region, but was significantly higher (p < 0.05) in the Tg-Saline mice compared with WT-Saline mice in the plaque-bearing region (Fig. 5C). TfRMAb-TNFR treatment did not alter TREM2-positive area compared with the Tg-Saline mice in the plaque-bearing region (Fig. 5C). As mentioned before, since 6E10 stains APP and all forms of Aβ, we specifically visualized β-sheet rich Aβ plaques by illuminating the brain tissue sections using 405 nm laser and found a significant reduction in the Tg-TfRMAb-TNFR mice compared with Tg-Saline mice (Fig. 5D; p < 0.01). Notably, the expression of TREM2 was significantly positively correlated with the Iba1 in the Aβ plaque-bearing brain regions and not in the plaque-free brain regions (Additional file 9: Fig. S8A, B). Further, the TREM2-positive area and the β-sheet rich Aβ plaque area were significantly positively correlated in the Aβ plaque-bearing brain regions (Additional file 9: Fig. S8C). Confocal microscopy therefore revealed that TREM2 was enriched within plaque-associated microglia, but not in microglia away from plaques. Interestingly, we found that Iba1 (Fig. 5E, F) and TREM2 (Fig. 5E and G) MFI was significantly (p < 0.05) higher in microglia associated with mature β-sheet rich Aβ plaques in Tg-TfRMAb-TNFR mice compared with Tg-Saline mice.

### No overt changes to the plasma metabolic panel with TfRMAb-TNFR

We performed a plasma metabolic panel on terminal plasma and studied the levels of 14 analytes (albumin, alkaline phosphatase (ALP), alanine transaminase (ALT), amylase, total bilirubin, blood urea nitrogen (BUN), calcium, phosphorus, creatinine, glucose, sodium, potassium, total protein, and globulin) (Additional file 10: Fig. S9A–N). TfRMAb-TNFR treatment of 3xTg-AD mice for 12 weeks slightly reduced albumin (Additional file 10: Fig. S9A; p < 0.01) and potassium (Additional file 10: Fig. S9L; p < 0.05), and increased phosphorus (Additional file 10: Fig. S9H; p < 0.001) compared to the Tg-Saline mice. All other parameters remained unchanged with TfRMAb-TNFR treatment (Additional file 10: Fig. S9O).

## Discussion

Using spatial proteomics, we show regional differences in the protein expression of 11-month-old female 3xTg-AD mice, with significant DEP in the Aβ plaque-bearing versus plaque-free hippocampal regions of the brain. The DEP were largely relevant to pathways involved in Aβ, tau, and neuroinflammation, with some changes in proteins involved in autophagy, neurodegeneration, and PD pathology. Chronic treatment with the brain-penetrating biologic TNF-α inhibitor, TfRMAb-TNFR, modulated DEP relevant to Aβ pathology, oligodendrocytes, microglial function, neurodegeneration, and autophagy, with most of the proteins being relevant to microglial function. Further analysis of the microglial response revealed clustering of these innate immune cells around larger Aβ deposits and an increase in larger Aβ deposits in Tg-TfRMAb-TNFR mice compared with Tg-Saline mice. This increased microglial localization around larger Aβ deposits was associated with reduced mature β-sheet-rich Aβ plaques and increased clustering of TREM2-positive microglia around mature Aβ plaques in the Tg-TfRMAb-TNFR mice compared with Tg-Saline mice. These results show a shift in the microglial response to the Aβ plaque environment of the Tg-TfRMAb-TNFR mice.

The 3xTg-AD mice harbor three mutations, APP with the Swedish mutation, PSEN1 with the M146V mutation, and microtubule-associated protein with the P301L mutation, resulting in Aβ plaque and tangle pathology [30, 51]. Besides, these mice are characterized by age-dependent inflammation, allowing them to recapitulate the key hallmarks of human AD which include Aβ, tau, and neuroinflammation [15, 30, 51, 52]. Over the years, delays in AD pathology have been observed in the 3xTg-AD mice, with recent characterization showing that Aβ pathology is present in the females around 18 months of age and is restricted to the subiculum [39]. Based on this, our study used younger female 3xTg-AD mice (age at the start: 8 months) to mimic a model of early intervention. In the current study, Aβ plaque and phosphorylated tau (AT8) were primarily observed in the subiculum of 11-month-old 3xTg-AD mice (Fig. 1B and Additional file 8: Fig. S7), consistent with previous observations [39], and we therefore focused our analysis on the hippocampal region.

First, due to the limited visible Aβ and tau pathology expected in the 11-month-old female 3xTg-AD mice [39], we decided to comprehensively investigate the AD-relevant protein signatures in the hippocampus of the 3xTg-AD mice in comparison with WT mice. Despite regional differences in the visible presence of the Aβ plaques, there were commonalities in the pathways modulated in the subiculum, CA2, and DG of the 3xTg-AD mice compared with the WT mice. More than 60% of the DEP in these regions were relevant to Aβ-processing, tau, and neuroinflammation pathways (Fig. 1G–J). These findings are consistent with the presence of these AD hallmarks in 3xTg-AD mice [35, 39, 53]. Our data also revealed some regional differences in protein expression in the 3xTg-AD mice compared with the WT mice. Out of the 64 proteins studied, 40% were differentially expressed in the subiculum and CA2 regions, and fewer (14%) were differentially regulated in the DG. Autophagy-relevant proteins (BAG3 and ATG12) were upregulated within the Aβ plaque-bearing subiculum region (Fig. 1G, Additional file 3: Fig. S2) compared with the WT mice. An inverse response was observed in the plaque-free CA2 hippocampal region, and autophagy-relevant proteins were largely downregulated compared with WT mice. Similarly, proteins relevant to neuronal health/neurodegeneration (synaptophysin, MAP2, neurofilament light) were downregulated in the plaque-free CA2 (Fig 1I, Additional file 5: Fig. S4) and DG (Fig. 1J, Additional file 6: Fig. S5) suggestive of an ongoing neurodegenerative process at sites distant from visible Aβ deposits.

To further understand if protein expression signatures differ in the Aβ plaque-bearing versus plaque-free microenvironments, protein expression was studied in the Aβ-plaque bearing versus Aβ-plaque free regions of the hippocampus of the 3xTg-AD mice. As mentioned above, as Aβ accumulation was observed in the subiculum, this region was selected as the plaque-bearing region for the analysis. The CA2 and DG regions of the hippocampus were selected as the plaque-free regions due to the absence of observable Aβ deposits. Interestingly, most of the DEP (⁓ 64%) showed decreased expression levels in the Aβ plaque-bearing versus plaque-free regions, and the expression profile strongly suggested an altered inflammatory and autophagy profile in the Aβ plaque microenvironment. The glial response in the Aβ plaque-bearing region was complex and while a reduction was observed in many key glial proteins (e.g., Iba1, GFAP, TMEM119, CD68, CD11b, CD163, Mertk) (Fig. 2A and C), some glial proteins were upregulated (SPP1, ITGAX, CSF1R) (Fig. 2A and B). These results are consistent with the unique gene expression profile observed in AD-associated glial cells, including microglia, compared to homeostatic glial cells, in humans and mice [54]. In this regard, disease associated microglia are enriched with genes associated with increased phagocytosis and activation including SPP1 and ITGAX, and the expression of homeostatic genes including TMEM119 and Mertk is reduced [54], consistent with the glial proteomic changes observed in the Aβ plaque-bearing versus plaque-free regions of the 3xTg-AD mice. Several autophagy markers (ATG5, LC3B, P62, and VPS35) were downregulated in the Aβ-plaque bearing region compared with the plaque-free regions. Autophagy plays an important role in Aβ clearance, and this process is impaired in AD [55]. Therefore, a decrease in autophagy may explain, at least in part, the increased Aβ load in the plaque-bearing regions of the 3xTg-AD mice. The upregulated protein expression profile in the Aβ plaque-bearing region was consistent with the APP and tau mutations found in the 3xTg-AD mice and included increased APP, Aβ1-42, and phosphorylated tau (Fig. 2A and B). This increase in Aβ1-42 appears to be driven by a reduction in Aβ degrading enzymes (neprilysin and IDE), that can increase Aβ, rather than an increase in APP processing by BACE1 or PSEN1 (which are involved in APP cleavage), both of which were reduced in the Aβ plaque-bearing regions. The Aβ plaque-bearing regions of the hippocampus were also marked by signs of neurodegeneration compared with the plaque-free regions as evident by a significant reduction in synaptophysin and neuronal nuclear protein (NeuN) (Fig. 2A and C), key markers of neuronal health [56–58], with a concomitant increase in the expression of neurofilament light and MBP (Fig. 2A and B); the latter are increased with neurodegeneration [59] and myelin injury [60, 61] in AD, respectively. Collectively, these spatial proteomic findings indicate that despite limited Aβ deposits observed by immunostaining, significant AD-relevant protein alterations are present in the hippocampus of the 11-month-old 3xTg-AD female mice. This mouse model which harbors the APP, PSEN1, and tau mutations shows dysregulation in glial, autophagy, and neurodegeneration pathways which are relevant to AD pathogenesis and progression.

Chronic treatment with the TNF-α inhibitor (TfRMAb-TNFR) modulated a number of these AD-relevant proteins in the hippocampus of the 3xTg-AD mice. TfRMAb-TNFR decreased the levels of BACE1 (Fig. 3B), the β-secretase involved in APP cleavage and Aβ generation [62]. This is in contrast to our previous work which showed no reduction in the expression of BACE1 in APP/PS1 mice after chronic TfRMAb-TNFR treatment [24], and this difference may be attributed to the measurement of only hippocampal BACE1 in the current study versus the measurement of whole brain BACE1 in our prior work. Further, TNF-α inhibition may alter different pathways in brains laden with low Aβ burden (current study) compared to brains with full-blown Aβ pathology (our prior work in aged APP/PS1 mice [24]). Interestingly, TfRMAb-TNFR treatment decreased the levels of neprilysin (Fig. 3A), an Aβ-degrading enzyme [63, 64] and the levels of the oligodendrocyte-related proteins, MBP (Fig. 3C), and olig2 (Fig. 3D). 3xTg-AD mice are known to show age- and Aβ-dependent myelin and oligodendrocyte disruption [65], and such changes may increase the levels of MBP and olig2 in AD brains [60, 61]. Notably, TNF-α can further potentiate these effects [66], and TNF-α blockade can limit demyelination and oligodendrocyte death [67]. These findings are in line with our data showing a reduction in these myelin and oligodendrocyte-associated proteins in the TfRMAb-TNFR-treated mice. Interestingly, most proteins modulated by TfRMAb-TNFR were related to microglia cells, the innate immune cells of the brain, and a diverse microglial response was observed following TfRMAb-TNFR treatment (Fig. 3E–H). TfRMAb-TNFR treatment altered the expression of disease-associated microglial markers SPP1, a conserved marker of activated/phagocytic microglia in AD mouse and patient brains [54], P2RX7, a purinergic receptor increased in AD brains [68], and ctsd, a lysosomal function marker [54]. We also found increased expression of the amyloid-responsive microglial marker CD163 with TfRMAb-TNFR treatment, suggesting an increased microglial response to Aβ deposits in the treated mice [69]. These results show that TfRMAb-TNFR mounts a significant innate immune response in the 3xTg-AD brains. Apart from a robust microglial response, TfRMAb-TNFR reduced proteins involved in neurodegeneration, gene expression, and autophagy. Mice treated with TfRMAb-TNFR had reduced phospho-α-synuclein, Tdp-43, and ULK1. These findings are relevant from the perspective of AD, since phospho-α-synuclein has been linked to AD pathology hallmarks including Aβ and tau [70], and Tdp-43, an intranuclear protein that regulates gene expression, is strongly associated with cognitive decline in AD patients [71]. The relevance of reduced ULK1, a critical regulator of autophagy [72], with TfRMAb-TNFR is unclear but ULK1 inhibition is associated with reduced axonal degeneration [73], which is in line with a reduction in MBP with TfRMAb-TNFR treatment; MBP is a marker of axonal degeneration [60, 61]. TfRMAb-TNFR treatment also reduced the levels of NRGN, a marker for synaptic dysfunction in AD [74, 75]. Taken together, these results show the multifactorial response of the TNF-α inhibitor in the hippocampus of the 3xTg-AD mice and point to a largely innate immune/microglial-centric mechanism of action in this mouse model [76].

Given the effect of TfRMAb-TNFR largely on pathways involved in Aβ production and clearance (BACE1 and neprilysin) and microglial phagocytosis (SPP1, CD163, P2RX7, Ctsd), we next sought to determine the significance of these findings. A reduction in BACE1 is expected to reduce amyloidogenic APP cleavage and reduce Aβ, while a reduction in neprilysin is expected to increase Aβ load. Accordingly, we did not see a reduction in the total Aβ load (using 6E10 which binds to all forms of Aβ and the precursor form) in mice treated with TfRMAb-TNFR (Fig. 4B). This is in contrast to our previous work which showed a robust reduction in 6E10-positive Aβ pathology in mouse models of amyloidosis [24, 25]. We did, however, observe increased microglial association with larger Aβ deposits (Fig. 4F, G) and reduced microglial association with smaller Aβ deposits (Fig. 4E) in the TfRMAb-TNFR-treated mice. TfRMAb-TNFR-treated mice also had a greater number of larger Aβ deposits (> 500 µm^2^) compared with saline-treated 3xTg-AD mice which had a greater number of smaller Aβ deposits (< 500 µm^2^) (Fig. 4H). These data suggest that TfRMAb-TNFR treatment alters microglia-Aβ interactions such that microglia preferentially associate with larger Aβ deposits in the TfRMAb-TNFR-treated mice. Though the definitive function of microglial clustering around Aβ lesions in AD brains is unclear, work in AD transgenic mice has shown that Aβ-plaque-associated microglia form a protective barrier around the plaque preventing neuritic dystrophy [77], and depletion of microglia reduced Aβ load in AD transgenic mice [78]. Therefore, the increased association of microglia with Aβ deposits may explain the larger Aβ deposits, that are shielded by microglia, in the TfRMAb-TNFR-treated 3xTg-AD mice compared with saline-treated 3xTg-AD mice.

Next, we examined the effect of TfRMAb-TNFR treatment on the mature β-sheet rich Aβ plaques instead of total Aβ load stained using the 6E10 antibody. Our goal was to determine if TfRMAb-TNFR treatment increased microglial association around mature Aβ plaques, as observed for 6E10-positive Aβ deposits, and if yes, elucidate the underlying mechanism responsible for the increased microglial clustering around mature Aβ plaques. For the latter, we studied the expression of TREM2, which plays an instrumental role in microglia clustering around Aβ plaques and mediating phagocytosis and degradation of Aβ by microglia in transgenic mouse models of AD [79–81]. We did not observe a difference in the Iba1-positive area in the hippocampus of mice treated with TfRMAb-TNFR (Fig. 5B), and this was independent of the presence of Aβ plaques. However, the TREM2-positive area was significantly higher only in the Aβ-plaque bearing hippocampal regions confirming the role of this receptor in microglial association with Aβ [82]. TfRMAb-TNFR treatment did not alter the total TREM2-positive area in the Aβ-plaque bearing hippocampal region (Fig. 5C). Interestingly, we found a significant reduction in the mature Aβ plaque-positive area in the TfRMAb-TNFR treated mice (Fig. 5D), which was associated with an increased association of Iba1-positive microglia and TREM2-positive microglia with mature Aβ plaques (Fig. 5F, G). The increased TREM2-positive microglial association with mature Aβ plaques may stimulate Aβ plaque phagocytosis thereby reducing mature Aβ plaque load or/and the increased microglial association around mature Aβ plaques may result in plaque compaction and size reduction. This is consistent with our work in the APP/PS1 mice showing increased Aβ-plaque associated phagocytic microglia with TfRMAb-TNFR [24]. One question remains as to why we do not see a reduction in 6E10-positive Aβ deposits despite an increase in microglial association. The reason for this is unclear but recent work shows that TREM2-positive microglia are not effective in clearing up larger Aβ deposits (> 100 µm^2^) [83], and since the TfRMAb-TNFR treated mice were laden with larger 6E10-positive Aβ deposits, TREM2 levels may not be sufficient to clear these larger 6E10-positive Aβ deposits. In the case of mature Aβ plaques, which are much fewer than the 6E10-positive Aβ deposits, the increased TREM2-positive microglial association may be sufficient to drive plaque compaction and/or phagocytosis. Overall, these findings suggest that TREM2-positive microglial clustering in the Aβ plaque-bearing regions may be involved in limiting β-sheet-rich Aβ plaques in the TfRMAb-TNFR-treated 3xTg-AD mice.

Though TfRMAb-TNFR resulted in an innate immune response (TREM2-positive microglial clustering) towards the β-sheet-rich Aβ plaques in the 3xTg-AD mice, we did not see significant changes in tau phosphorylation with the treatment. We measured several phosphorylated tau species, tau phosphorylated at ser202/thr205 (AT8) using immunofluorescence (Additional file 8: Fig. S7 and Additional file 11), and tau phosphorylated at ser199, thr231, ser396, ser404, and ser214 using Nanostring spatial proteomics (Additional file 12: Table S2), and none of these were altered in the hippocampus of the female 3xTg-AD mice following chronic TfRMAb-TNFR dosing. This was unexpected based on the significant association between innate immune response and tau phosphorylation [84] and our work in the PS19 mice that showed a significant reduction in the AT8-positive area with TfRMAb-TNFR treatment [26]. The exact reason for the discrepancy is unclear but may be explained by the 25-fold lower AT8-positive area in the current study compared with the PS19 mice used previously. Therefore, it is likely that the phosphorylated tau burden is very low to begin with. Additionally, tau phosphorylation may be independent of TNF-α and Aβ in the 3xTg-AD mouse model, and is consistent with the work done by Parachikova et al., wherein no change in tau phosphorylation was observed despite a reduction in Aβ and neuroinflammation in 3xTg-AD mice with anti-inflammatory treatment [85].

There are several areas of improvement in the current study. First, as mentioned earlier, AD phenotypes of 3xTg-AD mice have drifted since the model was developed [30]. There is only a limited number of Aβ and tau lesions at 12-months of age and an increased number of lesions at 18-months of age but primarily only in female 3xTg-AD mice [39]. Consistent with this, Aβ plaque deposits were primarily localized to the subiculum in the current study. The 11-month-old 3xTg-AD mice also showed sparse but significantly higher phospho-tau (ser202 and thr205, AT8) in the subiculum compared with the WT-Saline mice (Additional file 8: Fig. S7). However, as seen in Figs. 1 and 2, despite limited visible Aβ plaques and AT8 lesions, we identified several DEP in the 11-month-old 3xTg-AD female mice that are relevant to AD pathogenesis. Further, our design of comparing the plaque-free and plaque-bearing regions in the current study is simplified and based solely on the visual presence of Aβ. Notably, the Aβ plaque distribution pattern herein, although low, follows a distribution pattern consistent with other studies in 3xTg-AD mice [35, 39] and mimics the increased susceptibility of the subiculum to AD pathology in humans, and tau pathology follows Aβ pathology in the 3xTg-AD mice [86, 87]. Therefore, while changes observed by us are likely driven by Aβ, the impact of other factors including transgene expression levels and tissue anatomy on protein expression in these regions cannot be ruled out. Second, though we saw a significant reduction in mature Aβ plaques with TfRMAb-TNFR treatment, no reduction in 6E10-positive Aβ load was observed. Similarly, we did not see a significant reduction in tau phosphorylation. Our previous work in aged and young male APP/PS1 mice showed a significant reduction in 6E10-positive Aβ load [24, 25], and our prior work in the female PS19 mice showed a significant reduction in phospho-tau (ser202 and thr205, AT8) with chronic TfRMAb-TNFR dosing [26]. There are a few possible reasons for this discrepancy. Our previous work used male APP/PS1 mice and the current study used female 3xTg-AD mice. Further, the Aβ and phospho-tau load in the current study was low and localized primarily to the subiculum, compared with the APP/PS1 and PS19 mice that show widespread Aβ and phospho-tau deposits in the brain, respectively. Therefore, studies in older 3xTg-AD mice with greater Aβ and tau pathology may be needed. Third, we used normal saline instead of the vehicle as the control. However, we do not expect the vehicle to produce therapeutic effects based on our prior work showing no effect of TfRMAb (without the TNFR domain) formulated in a similar vehicle (10 mM sodium acetate, 150 mM NaCl, and 0.01% polysorbate 80, pH = 6) in APP/PS1 mice [24]. Fourth, the effect of TfRMAb-TNFR on WT mice was not studied herein, which can help outline any possible untoward effects in healthy mice. Notably, TfRMAb-TNFR does not alter the plasma metabolic panel (Additional file 10: Fig. S9) and weight and locomotion (Additional file 13: Fig. S10A, and D-E and Additional file 11) in the 3xTg-AD mice, and hematology profile in the APP/PS1 [24] and PS19 [26] mice after chronic dosing. Fifth, it should be noted that the 6E10 antibody used herein recognizes Aβ with an intact N-terminal but also reacts with the precursor form and catabolic fragments of Aβ [88]. While we recognize that there is no ideal antibody to detect total Aβ, using an additional antibody, for example the 4G8, may be a better approach to quantify Aβ. Sixth, though Nanostring DSP offers the advantage of being both quantitative and spatial, compared with other traditional methods including Western blotting and immunostaining which are semiquantitative and/or do not offer spatial visualization, there are limitations to the use of Nanostring DSP technology including the limited size of the ROI and inability to provide single-cell resolution. Therefore, future studies using multiple approaches to detect the target protein (e.g., Western blotting, immunostaining, and NanoString DSP) will increase the rigor of the data. Finally, the 3xTg-AD mice in the current study did not display impaired performance in the Y-maze test but did show poor outcomes during the nest building test (Additional file 13: Fig. S10B-C and F-G and Additional file 11). TfRMAb-TNFR treatment, however, did not improve the nest-building outcomes in the current study. It is conceivable that in situations characterized with low Aβ load and tau pathology, as seen in the 11-month-old 3xTg-AD mice, the TfRMAb-TNFR modulates several pathways involved in Aβ formation, microglial function, neurodegeneration, gene expression and autophagy, the positive impact of which becomes evident as the disease progresses. Longitudinal studies in older 3xTg-AD mice may help clarify this.

## Conclusion

**Fig. 6:**
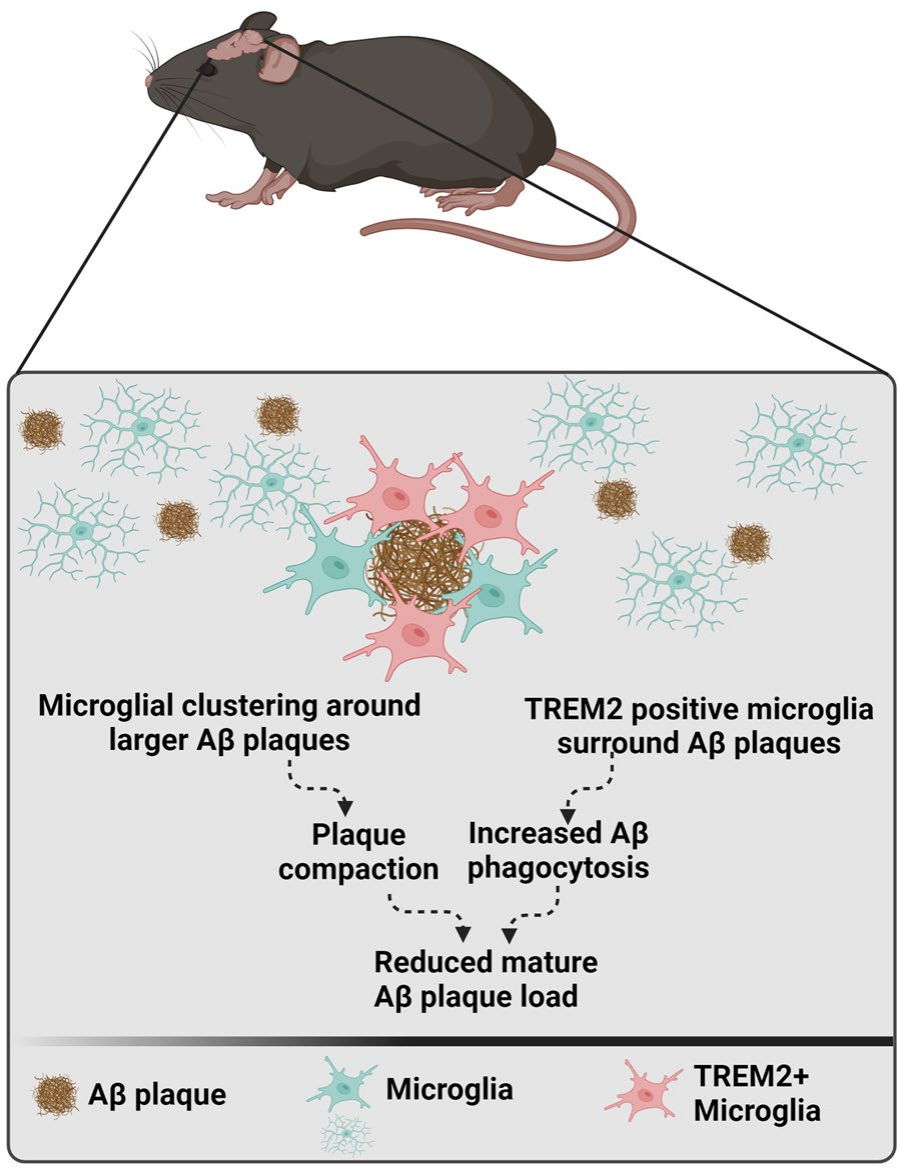
Schematic showing the mechanism of action of TfRMAb-TNFR in Aβ plaque reduction. TfRMAb-TNFR increases TREM2-positive microglial clustering around Aβ plaques, which is associated with a reduction in mature Aβ plaque load in the 3xTg-AD mice. No change in tau pathology is observed with TfRMAb-TNFR in this mouse model. Figure was prepared using BioRender.com

In conclusion, using spatial proteomics, we identified several AD-relevant differentially regulated proteins that modulate Aβ, tau, glial, autophagy, and/or neurodegeneration pathways in the hippocampus of 11-month-old female 3xTg-AD mice, despite very limited visible Aβ and tau pathology. Chronic treatment with a brain-penetrant TNF-α inhibitor increased TREM2-positive microglial clustering around Aβ plaques, which was associated with a reduction in mature Aβ plaque load (Fig. 6) without affecting tau pathology. Overall, our studies suggest a largely innate immune/microglial-centric mechanism of action of the brain-penetrant TNF-α inhibitor on Aβ pathology in the 3xTg-AD female mice.

### Supplementary Information

**Additional file 1: Table S1.** Number of outliers removed from Figs. 3 to 5.

**Additional file 2: Fig. S1.** Representative images from NanoString GeoMX DSP platform of a sagittal brain section from WT-Saline mice showing the circular hippocampal regions of interest (ROIs) (A). The red-boxed region in the brain section image in the left panel of A, taken from the Allen Institute, represents the hippocampus. The circled ROIs in the right panel of A are shown as high-resolution images for subiculum upper (dorsal subiculum) (B), CA2 (C), subiculum lower (ventral subiculum) (D), and DG (E) subregions labeled with the four morphology markers: Aβ (yellow), Iba1 (red), GFAP (green), and nuclear marker SYTO13 (blue). Scale bar = 500 μm in A and 50 μm in B–E.

**Additional file 3: Fig. S2.** Bar plots representing fold change (FC) values expressed in log2 FC showing protein expression in 3xTg-AD mice relative to WT-Saline mice in the upper subiculum region (up-regulation (A) and down-regulation (B)) of the hippocampus (n = 6/group). *p < 0.05.

**Additional file 4: Fig. S3.** Bar plots representing fold change (FC) values expressed in log2 FC showing protein expression in 3xTg-AD mice relative to WT-Saline mice in the lower subiculum region (up-regulation (A) and down-regulation (B)) of the hippocampus (n = 6/group). *p < 0.05.

**Additional file 5: Fig. S4.** Bar plots representing fold change (FC) values expressed in log2 FC showing protein expression in 3xTg-AD mice relative to WT-Saline mice in the CA2 region (up-regulation (A) and down-regulation (B)) of the hippocampus (n = 6/group). *p < 0.05.

**Additional file 6: Fig. S5.** Bar plots representing fold change (FC) values expressed in log2 FC showing protein expression in 3xTg-AD mice relative to WT-Saline mice in the dentate gyrus (DG) region (up-regulation (A) and down-regulation (B)) of the hippocampus (n = 6/group). *p < 0.05.

**Additional file 7: Fig. S6.** Effect of TfRMAb-TNFR on total 6E10 count/µm^2^ of the entire hippocampus (A) and intraneuronal 6E10-positive area as a % of tissue area in the plaque-bearing subiculum (B) of 3xTg-AD mice. The data are shown as Mean ± SEM for Tg-Saline (n = 11) and Tg-TfRMAb-TNFR (n = 11) mice. Data were analyzed using the unpaired t-test in A and Mann–Whitney U test in B. ns = not significant for the indicated comparisons.

**Additional file 8: Fig. S7.** Effect of TfRMAb-TNFR on AT8-positive area in the hippocampus of 3xTg-AD mice. Representative confocal images of AT8-positive immunofluorescence staining in the subiculum of 3xTg-AD mice with or without TfRMAb-TNFR treatment (A). Scale bar  = 25 μm in A. AT8-positive area (B). The data are shown as Mean ± SEM for WT-Saline (n = 5), Tg-Saline (n = 10), and Tg-TfRMAb-TNFR (n = 9) mice. Data were analyzed using the one-way ANOVA with Holm Sidak’s post-hoc test compared to Tg-Saline mice. **p < 0.01 and ns = not significant for the indicated comparisons.

**Additional file 9: Fig. S8.** Scatter plots show the correlation between Iba1 and TREM2% positive area in the plaque-bearing (subiculum) (A) and plaque-free (CA2) hippocampus (B), and mature Aβ plaques and TREM2% positive area in the plaque-bearing hippocampus (C) of 3xTg-AD mice by the Pearson correlation coefficient. Bar graph showing no difference in the TREM2 area when normalized to Iba1 area in the plaque-bearing and plaque-free hippocampus (D) consistent with TREM2-positive area % data shown in Figure 5C.  Significantly higher 6E10-associated microglia area normalized to 6E10 area with TfRMAb-TNFR in the plaque-bearing subiculum (E) consistent with Figure 4D which shows 6E10-associated microglial MFI. The data are shown as Mean ± SEM for WT-Saline (n = 9), Tg-Saline (n = 10–11), and Tg-TfRMAb-TNFR (n = 8–11) mice. Data were analyzed using the two-way repeated measures ANOVA with Holm Sidak’s post-hoc test in D, and unpaired t-test in E. *p < 0.05 and **p < 0.01 for the indicated comparisons.

**Additional file 10: Fig. S9.** Plasma metabolic panel of 3xTg-AD mice with or without TfRMAb-TNFR treatment. Albumin (A), alkaline phosphatase (ALP) (B), alanine transaminase (ALT) (C), amylase (D), total bilirubin (E), BUN (F), calcium (G), phosphorus (H), creatinine (I), glucose (J), sodium (K), potassium (L), total protein (M), globulin (N). The data are shown as Mean ± SEM for WT-Saline (n = 8–9), Tg-Saline (n = 10–11), and Tg-TfRMAb-TNFR (n = 10–11) mice. Data are reported as % of Tg-Saline values and were analyzed using the one-way ANOVA with Holm Sidak’s post-hoc test. The heat map of p values (O). *p < 0.05, **p < 0.01, ***p < 0.001 for the indicated comparisons.

**Additional file 11.** Materials and methods.

**Additional file 12: Table S2.** List of differentially and non-differentially expressed proteins in Tg-TfRMAb-TNFR compared with Tg-Saline 3xTg-AD mice.

**Additional file 13: Fig. S10.** Weights, open field, Y-maze, and nest building test in 3xTg-AD mice with or without TfRMAb-TNFR treatment. Body weight of animals during the study (A). Representative trajectory maps of mouse movement in the Y-maze test (B), and discrimination index, latency to novel arm, and % entries in the novel arm (C). Representative trajectory maps showing the mouse activity in the open-field test (D), and total distance traveled and mean speed (E) in the open-field arena. Representative images of nests built by 3xTg-AD mice compared with age-matched WT mice (F), and nesting scores and amount of untorn nestlet (G). The data are shown as Mean ± SEM for WT (n = 9), Tg-Saline (n = 11), and Tg-TfRMAb-TNFR (n = 11) per group. The data were analyzed using two-way repeated measures ANOVA in A, one-way ANOVA with Holm Sidak’s post-hoc test in C and E, and Kruskal–Wallis test with Dunn’s post-hoc test in G. *p < 0.05.

## Data Availability

The datasets used and/or analyzed during the current study are available from the corresponding author on reasonable request.

## Abbreviations

ALT: Alanine transaminase
ALP: Alkaline phosphatase
AD: Alzheimer’s disease
Aβ: Amyloid beta
BACE1: Beta-Secretase 1
bTNFIs: Biologic TNF-α inhibitors
BUN: Blood urea nitrogen
BSA: Bovine serum albumin
DG: Dentate gyrus
DEP: Differentially expressed proteins
FC: Fold-change
GFAP: Glial fibrillary acidic protein
MFI: Mean fluorescent intensity
TfRMAb: Mouse transferrin receptor antibody
MBP: Myelin Basic Protein
NRGN: Neurogranin
Oligo2: Oligodendrocyte transcription factor 2
PD: Parkinson’s disease
PBS: Phosphate buffered saline
ROIs: Regions of interest
RT: Room temperature
Tdp43: TAR DNA-binding protein 43
TREM2: Triggering receptor expressed on myeloid cells-2
TNF-α: Tumor necrosis factor alpha
ULK1: Unc-51-like kinase 1
