# Supplementary material for: Modulation of hippocampal protein expression by a brain penetrant biologic TNF-α inhibitor in the 3xTg Alzheimer’s disease mice
Source: J Transl Med. 2024 Mar 18;22:291. doi: 10.1186/s12967-024-05008-x (PMC10946165; doi:10.1186/s12967-024-05008-x)
Supplement: Supplementary file 1 — Additional file 1: Table S1. Number of outliers removed from Figs. 3 to 5. [file 12967_2024_5008_MOESM1_ESM.docx]

**Table. S1. Number of outliers removed from Figure 3 to Figure 5.**

| **Figures** | **Original number of mice** | **Group** | **Outliers** |
| --- | --- | --- | --- |
| Figure 3A | 6 | Tg-Saline | 1 |
|  | 6 | Tg-TfRMAb-TNFR | - |
| Figure 3B | 6 | Tg-Saline | 1 |
|  | 6 | Tg-TfRMAb-TNFR | 1 |
| Figure 3C | 6 | Tg-Saline | 1 |
|  | 6 | Tg-TfRMAb-TNFR | 1 |
| Figure 3D | 6 | Tg-Saline | 1 |
|  | 6 | Tg-TfRMAb-TNFR | - |
| Figure 3E | 6 | Tg-Saline | - |
|  | 6 | Tg-TfRMAb-TNFR | 1 |
| Figure 3F | 6 | Tg-Saline | - |
|  | 6 | Tg-TfRMAb-TNFR | 1 |
| Figure 3G | 6 | Tg-Saline | 1 |
|  | 6 | Tg-TfRMAb-TNFR | 1 |
| Figure 3H | 6 | Tg-Saline | - |
|  | 6 | Tg-TfRMAb-TNFR | 1 |
| Figure 3I | 6 | Tg-Saline | 1 |
|  | 6 | Tg-TfRMAb-TNFR | 1 |
| Figure 3J | 6 | Tg-Saline | 1 |
|  | 6 | Tg-TfRMAb-TNFR | 1 |
| Figure 3K | 6 | Tg-Saline | 1 |
|  | 6 | Tg-TfRMAb-TNFR | 1 |
| Figure 3L | 6 | Tg-Saline | - |
|  | 6 | Tg-TfRMAb-TNFR | 1 |
| Figure 4D | WT-Saline (9)  Tg-Saline (10)  Tg-TfRMAb-TNFR (10) | WT-Saline  Tg-Saline  Tg-TfRMAb-TNFR | -  -  1 |
| Figure 5C-D | WT-Saline (9)  Tg-Saline (11)  Tg-TfRMAb-TNFR (11) | WT-Saline  Tg-Saline  Tg-TfRMAb-TNFR | -  **-**  1 |
