# Supplementary figures and images for: Modulation of hippocampal protein expression by a brain penetrant biologic TNF-α inhibitor in the 3xTg Alzheimer’s disease mice
Source: J Transl Med. 2024 Mar 18;22:291. doi: 10.1186/s12967-024-05008-x (PMC10946165; doi:10.1186/s12967-024-05008-x)

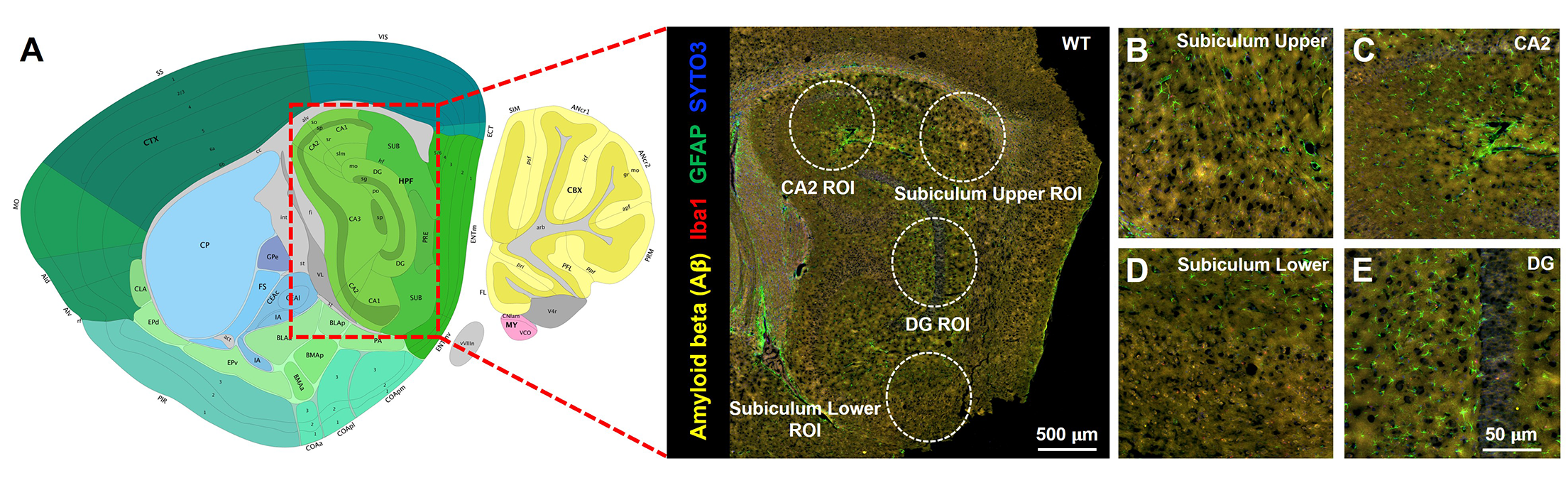

Supplement: Supplementary file 2 — Additional file 2: Fig. S1. Representative images from NanoString GeoMX DSP platform of a sagittal brain section from WT-Saline mice showing the circular hippocampal regions of interest (ROIs) (A). The red-boxed region in the brain section image in the left panel of A, taken from the Allen Institute, represents the hippocampus. The circled ROIs in the right panel of A are shown as high-resolution images for subiculum upper (dorsal subiculum) (B), CA2 (C), subiculum lower (ventral subiculum) (D), and DG (E) subregions labeled with the four morphology markers: Aβ (yellow), Iba1 (red), GFAP (green), and nuclear marker SYTO13 (blue). Scale bar = 500 μm in A and 50 μm in B–E. [file 12967_2024_5008_MOESM2_ESM.tif]

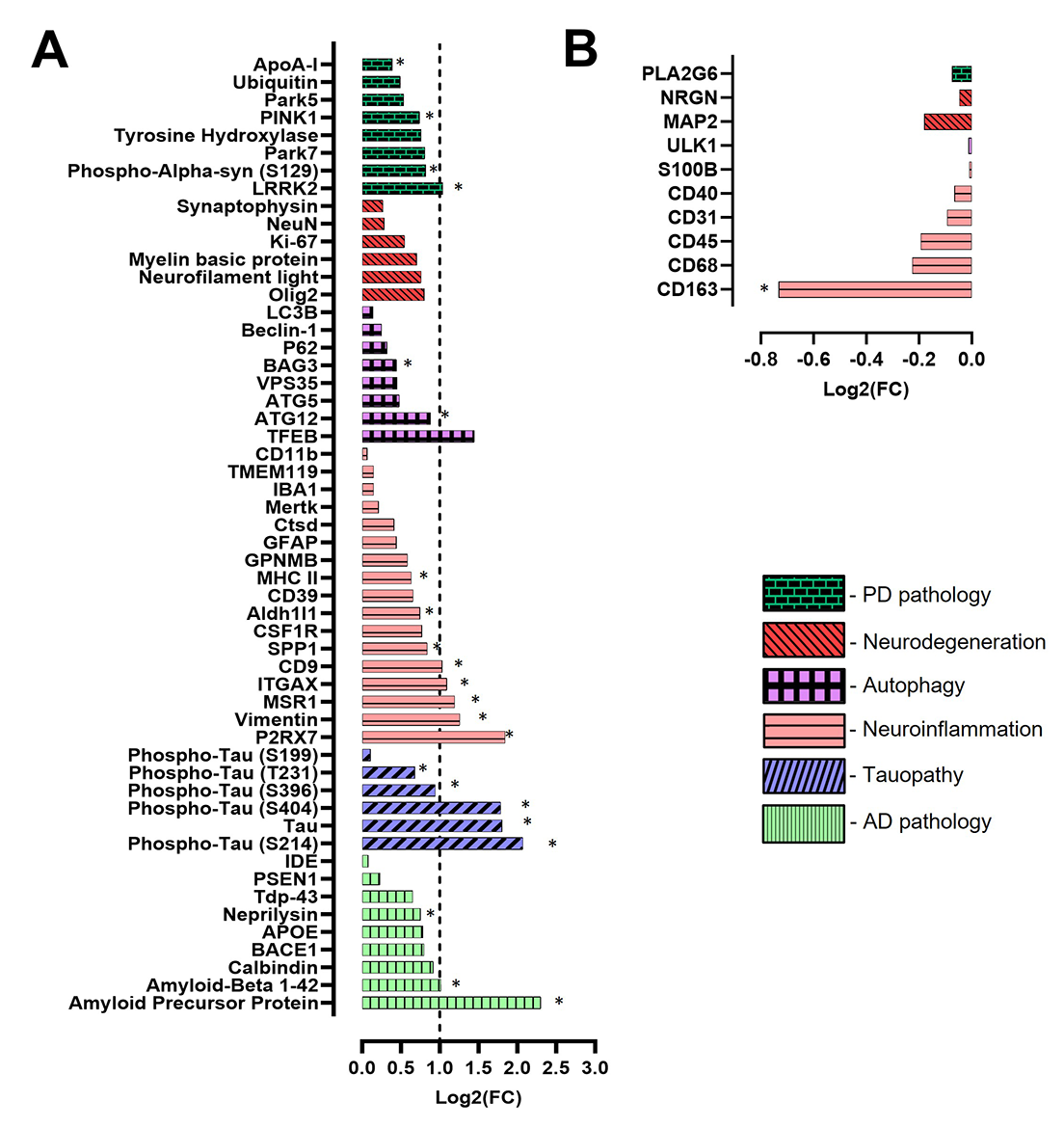

Supplement: Supplementary file 3 — Additional file 3: Fig. S2. Bar plots representing fold change (FC) values expressed in log2 FC showing protein expression in 3xTg-AD mice relative to WT-Saline mice in the upper subiculum region (up-regulation (A) and down-regulation (B)) of the hippocampus (n = 6/group). *p < 0.05. [file 12967_2024_5008_MOESM3_ESM.tif]

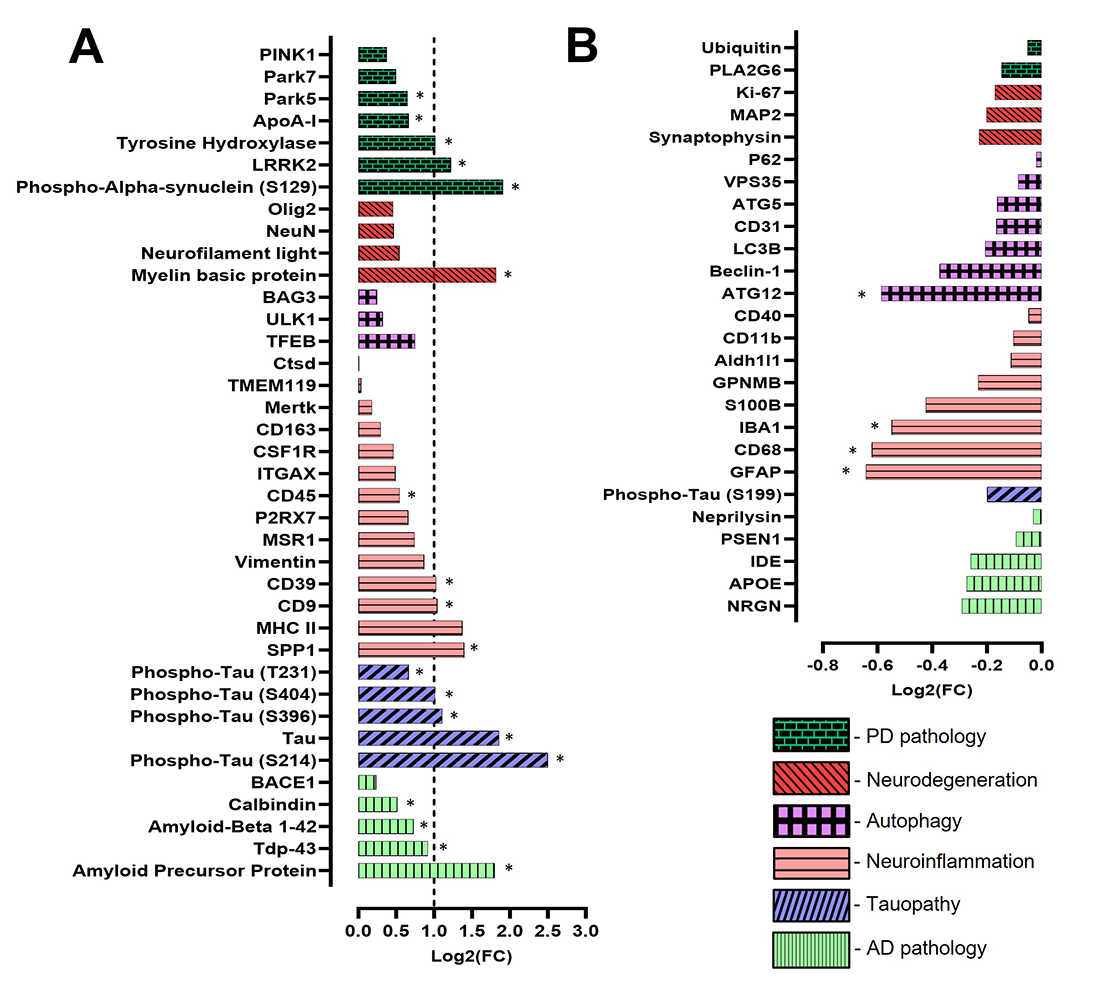

Supplement: Supplementary file 4 — Additional file 4: Fig. S3. Bar plots representing fold change (FC) values expressed in log2 FC showing protein expression in 3xTg-AD mice relative to WT-Saline mice in the lower subiculum region (up-regulation (A) and down-regulation (B)) of the hippocampus (n = 6/group). *p < 0.05. [file 12967_2024_5008_MOESM4_ESM.tif]

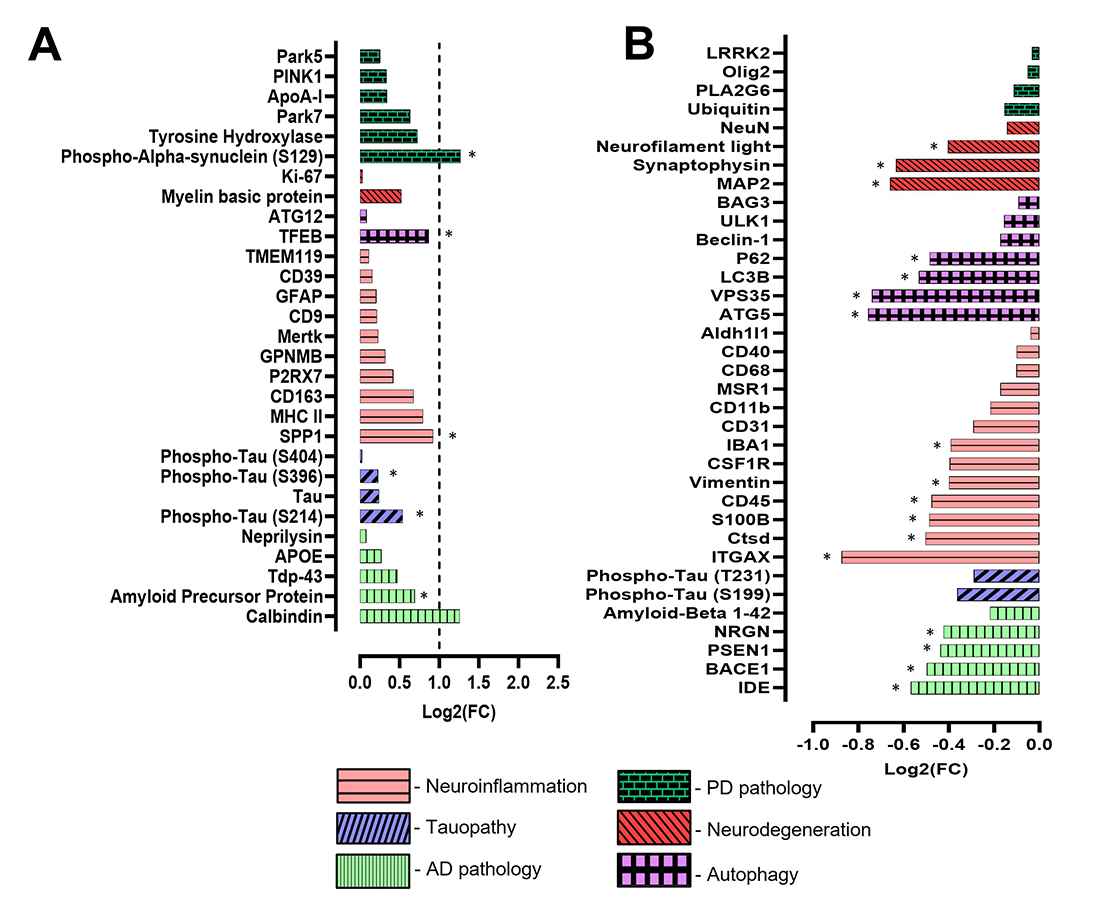

Supplement: Supplementary file 5 — Additional file 5: Fig. S4. Bar plots representing fold change (FC) values expressed in log2 FC showing protein expression in 3xTg-AD mice relative to WT-Saline mice in the CA2 region (up-regulation (A) and down-regulation (B)) of the hippocampus (n = 6/group). *p < 0.05. [file 12967_2024_5008_MOESM5_ESM.tif]

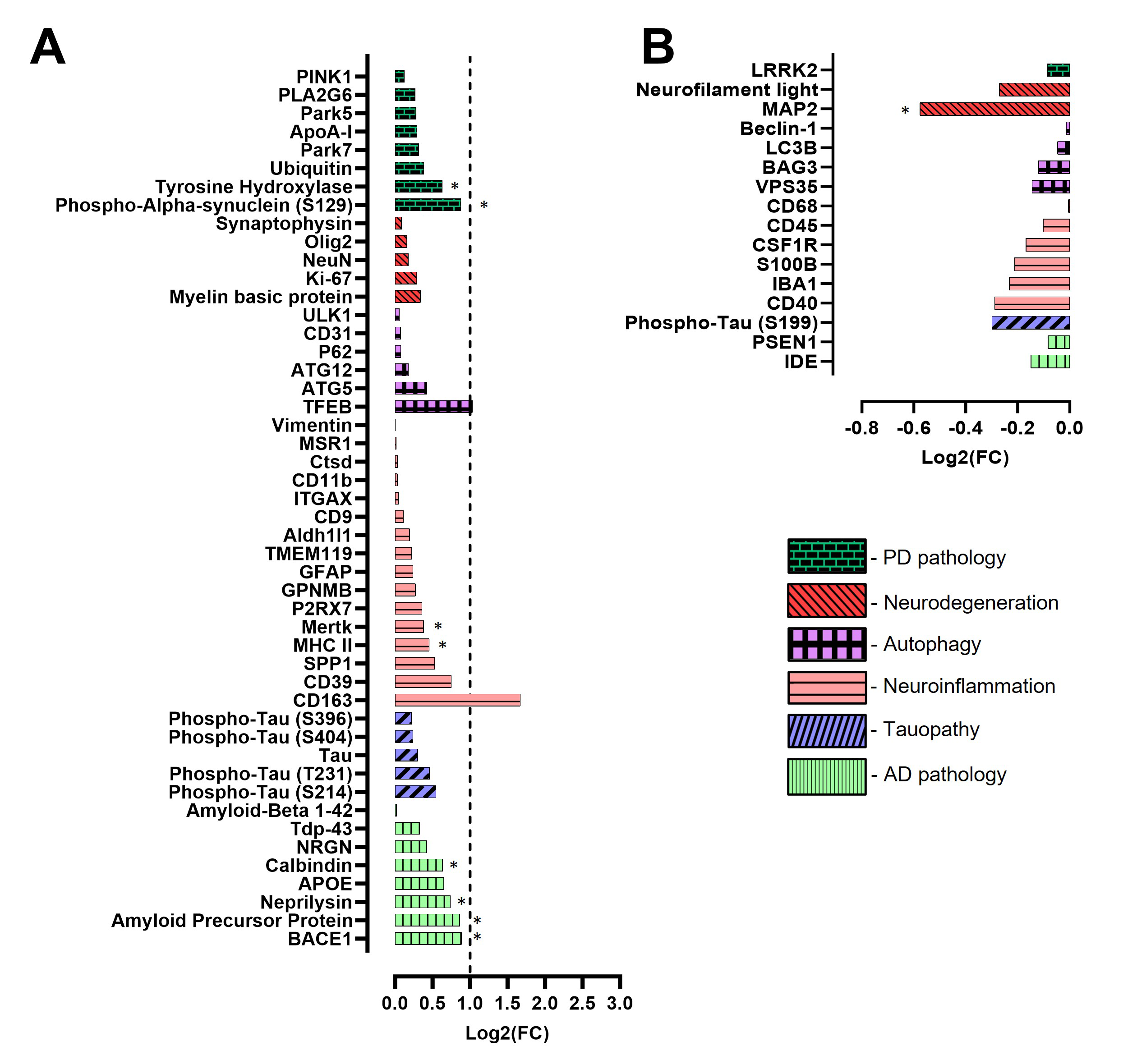

Supplement: Supplementary file 6 — Additional file 6: Fig. S5. Bar plots representing fold change (FC) values expressed in log2 FC showing protein expression in 3xTg-AD mice relative to WT-Saline mice in the dentate gyrus (DG) region (up-regulation (A) and down-regulation (B)) of the hippocampus (n = 6/group). *p < 0.05. [file 12967_2024_5008_MOESM6_ESM.tif]

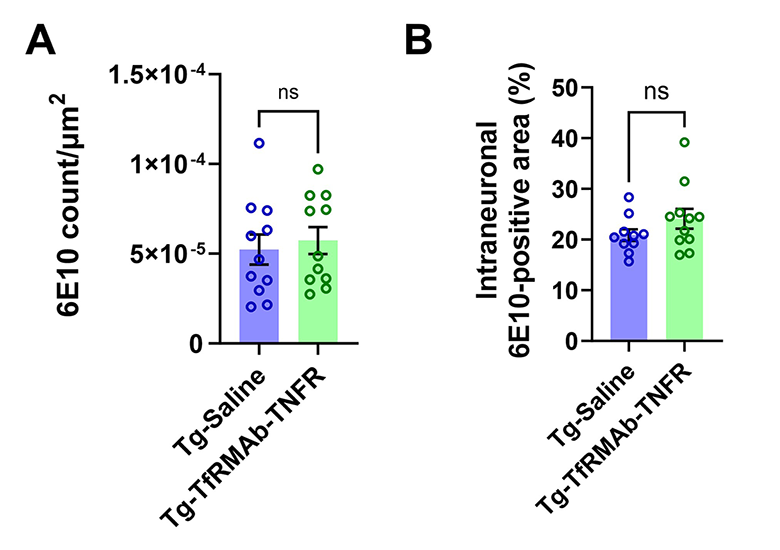

Supplement: Supplementary file 7 — Additional file 7: Fig. S6. Effect of TfRMAb-TNFR on total 6E10 count/µm2 of the entire hippocampus (A) and intraneuronal 6E10-positive area as a % of tissue area in the plaque-bearing subiculum (B) of 3xTg-AD mice. The data are shown as Mean ± SEM for Tg-Saline (n = 11) and Tg-TfRMAb-TNFR (n = 11) mice. Data were analyzed using the unpaired t-test in A and Mann–Whitney U test in B. ns = not significant for the indicated comparisons. [file 12967_2024_5008_MOESM7_ESM.tif]

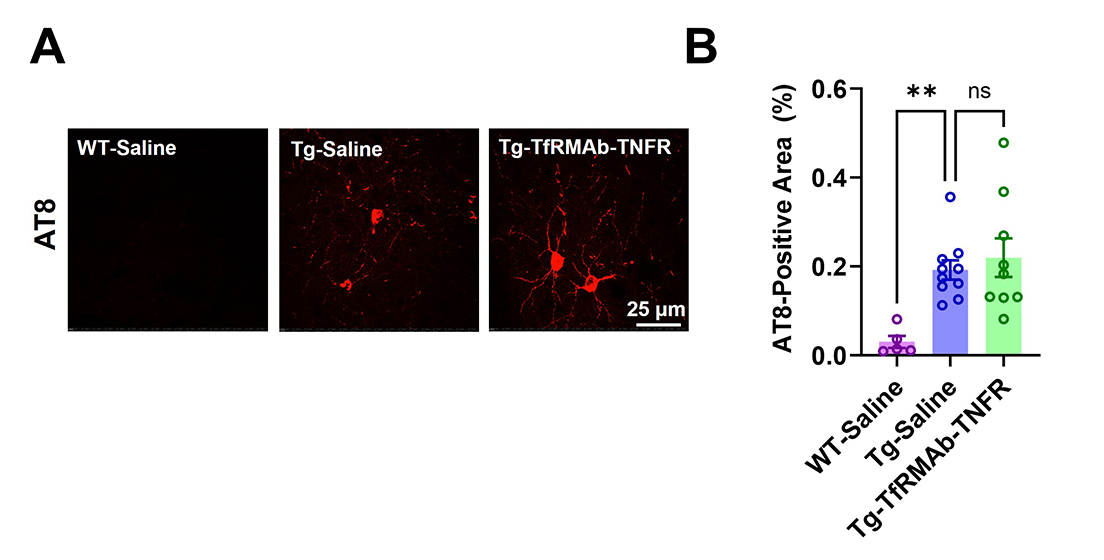

Supplement: Supplementary file 8 — Additional file 8: Fig. S7. Effect of TfRMAb-TNFR on AT8-positive area in the hippocampus of 3xTg-AD mice. Representative confocal images of AT8-positive immunofluorescence staining in the subiculum of 3xTg-AD mice with or without TfRMAb-TNFR treatment (A). Scale bar = 25 μm in A. AT8-positive area (B). The data are shown as Mean ± SEM for WT-Saline (n = 5), Tg-Saline (n = 10), and Tg-TfRMAb-TNFR (n = 9) mice. Data were analyzed using the one-way ANOVA with Holm Sidak’s post-hoc test compared to Tg-Saline mice. **p < 0.01 and ns = not significant for the indicated comparisons. [file 12967_2024_5008_MOESM8_ESM.tif]

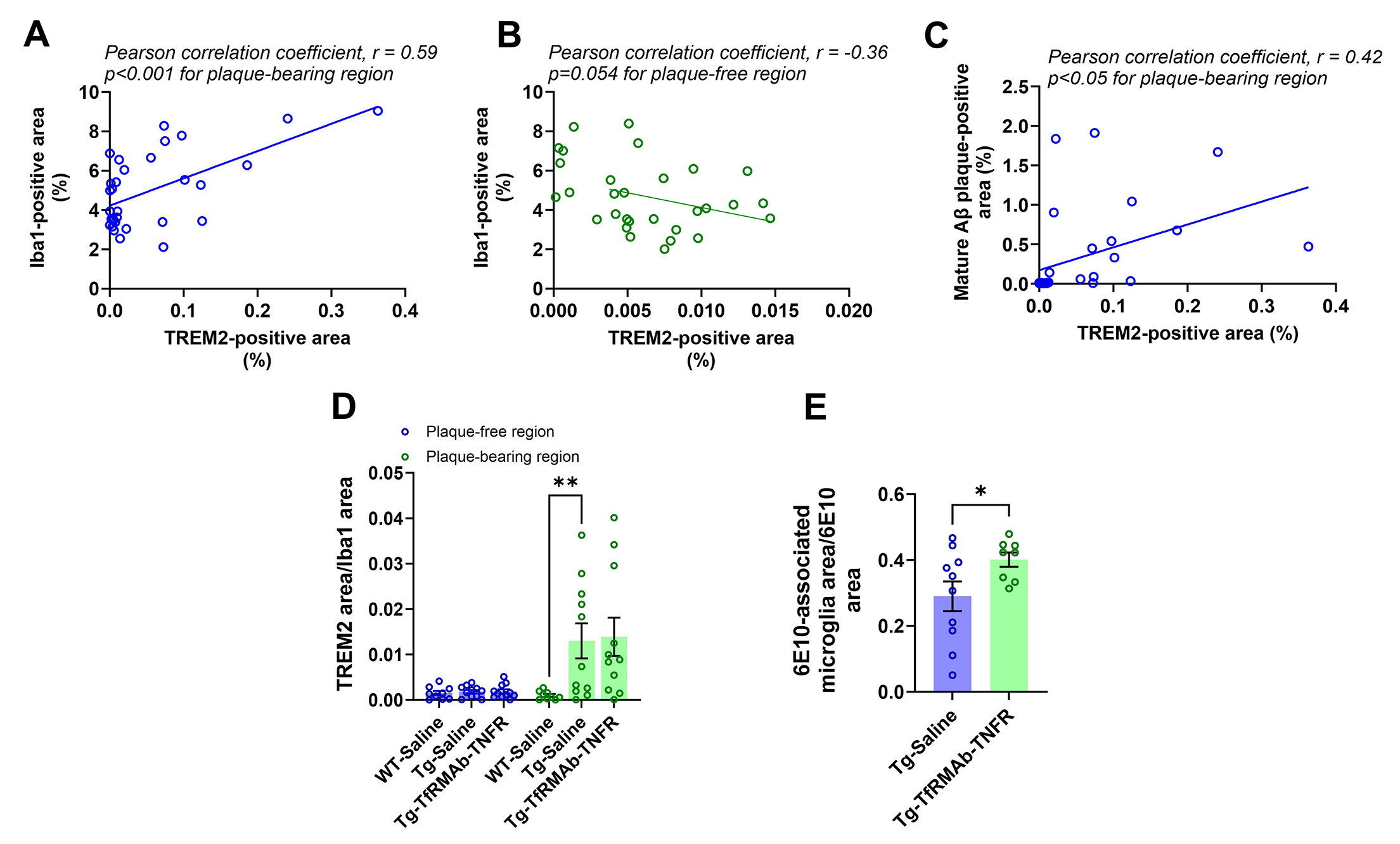

Supplement: Supplementary file 9 — Additional file 9: Fig. S8. Scatter plots show the correlation between Iba1 and TREM2% positive area in the plaque-bearing (subiculum) (A) and plaque-free (CA2) hippocampus (B), and mature Aβ plaques and TREM2% positive area in the plaque-bearing hippocampus (C) of 3xTg-AD mice by the Pearson correlation coefficient. Bar graph showing no difference in the TREM2 area when normalized to Iba1 area in the plaque-bearing and plaque-free hippocampus (D) consistent with TREM2-positive area % data shown in Figure 5C. Significantly higher 6E10-associated microglia area normalized to 6E10 area with TfRMAb-TNFR in the plaque-bearing subiculum (E) consistent with Figure 4D which shows 6E10-associated microglial MFI. The data are shown as Mean ± SEM for WT-Saline (n = 9), Tg-Saline (n = 10–11), and Tg-TfRMAb-TNFR (n = 8–11) mice. Data were analyzed using the two-way repeated measures ANOVA with Holm Sidak’s post-hoc test in D, and unpaired t-test in E. *p < 0.05 and **p < 0.01 for the indicated comparisons. [file 12967_2024_5008_MOESM9_ESM.tif]

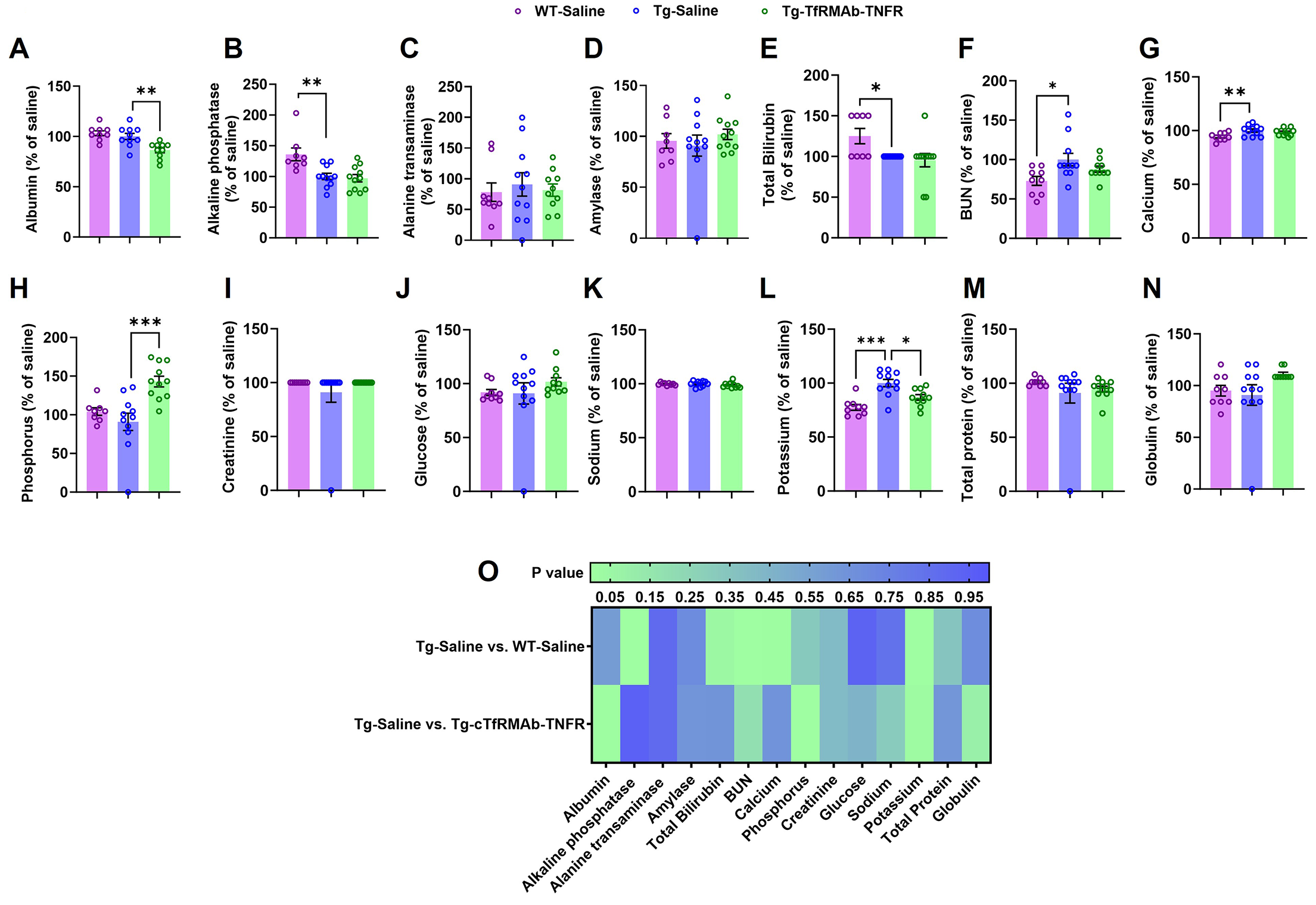

Supplement: Supplementary file 10 — Additional file 10: Fig. S9. Plasma metabolic panel of 3xTg-AD mice with or without TfRMAb-TNFR treatment. Albumin (A), alkaline phosphatase (ALP) (B), alanine transaminase (ALT) (C), amylase (D), total bilirubin (E), BUN (F), calcium (G), phosphorus (H), creatinine (I), glucose (J), sodium (K), potassium (L), total protein (M), globulin (N). The data are shown as Mean ± SEM for WT-Saline (n = 8–9), Tg-Saline (n = 10–11), and Tg-TfRMAb-TNFR (n = 10–11) mice. Data are reported as % of Tg-Saline values and were analyzed using the one-way ANOVA with Holm Sidak’s post-hoc test. The heat map of p values (O). *p < 0.05, **p < 0.01, ***p < 0.001 for the indicated comparisons. [file 12967_2024_5008_MOESM10_ESM.tif]

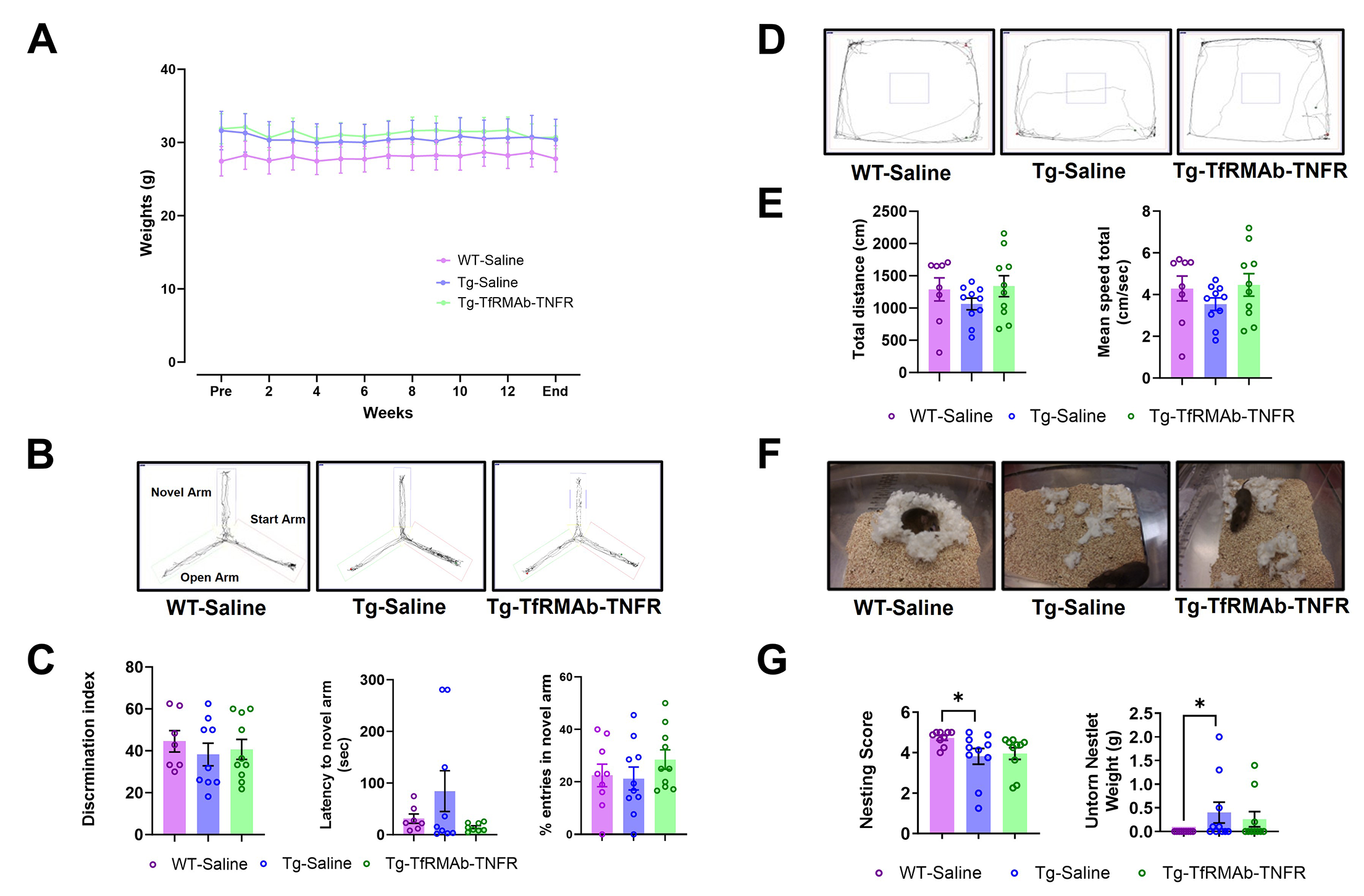

Supplement: Supplementary file 13 — Additional file 13: Fig. S10. Weights, open field, Y-maze, and nest building test in 3xTg-AD mice with or without TfRMAb-TNFR treatment. Body weight of animals during the study (A). Representative trajectory maps of mouse movement in the Y-maze test (B), and discrimination index, latency to novel arm, and % entries in the novel arm (C). Representative trajectory maps showing the mouse activity in the open-field test (D), and total distance traveled and mean speed (E) in the open-field arena. Representative images of nests built by 3xTg-AD mice compared with age-matched WT mice (F), and nesting scores and amount of untorn nestlet (G). The data are shown as Mean ± SEM for WT (n = 9), Tg-Saline (n = 11), and Tg-TfRMAb-TNFR (n = 11) per group. The data were analyzed using two-way repeated measures ANOVA in A, one-way ANOVA with Holm Sidak’s post-hoc test in C and E, and Kruskal–Wallis test with Dunn’s post-hoc test in G. *p < 0.05. [file 12967_2024_5008_MOESM13_ESM.tif]
