# Supplementary material for: Modulation of hippocampal protein expression by a brain penetrant biologic TNF-α inhibitor in the 3xTg Alzheimer’s disease mice
Source: J Transl Med. 2024 Mar 18;22:291. doi: 10.1186/s12967-024-05008-x (PMC10946165; doi:10.1186/s12967-024-05008-x)
Supplement: Supplementary file 11 — Additional file 11. Materials and methods. [file 12967_2024_5008_MOESM11_ESM.doc]

**Additional Materials and Methods**

**Open field test**

All the behavior tests were performed at the end of treatment when the mice were 11 months old, and the open field test was performed as previously described [1]. The open field box was 72 cm long x 72 cm wide x 36 cm high with white surrounding walls and a white bottom. Before testing, mice were transferred to the behavior room to adapt to the environment for at least 30 minutes. Then, the mice were placed in the open field arena and allowed to move freely. Mouse movements were recorded with a digital camera for 5 minutes and the total distance and the mean speed were quantified using SMART Video Tracking Software (Panlab, Harvard Apparatus). The arena was cleaned with 70% ethanol between tests to prevent odor cues.

**Y-Maze**

The Y-maze test was performed as previously described [1] using an apparatus consisting of a three-arm horizontal maze (30 cm long, 10 cm wide, and 25 cm high). The mice were habituated to the maze for 8 minutes with the novel arm closed. Thirty minutes after habituation, the mice were placed back in the start arm and allowed to explore all three arms. The discrimination index (the number of entries in the novel arm divided by the total number of entries in the novel and familiar arm), latency to the novel arm, and percentage of entries in the novel arm were quantified by the SMART Video Tracking Software (Panlab, Harvard Apparatus). The Y-maze was wiped with 70% ethanol between each animal trail.

**Nest building**

Nest building was used as a measure of overall well-being in mice. All mice were allocated to a single cage containing pre-weighed nestlets (approximately 2.5 g; Newco Specialty). The next day, the weight of the untorn nestlet was recorded. A 5-point score scale was used to score the nests built as follows: 1 – No shredding no nest, majority of nestlet (>90%) is still intact, 2 – a noticeable portion of the nestlet is torn (50-90% remaining intact), 3 – majority of the nestlet is torn (<50% remaining intact), 4 – >90% of the nestlet is shredded and flat nest, and 5 – all shredded and a perfect nest (>90% of the nestlet is torn). All mice were tested at the age of 11 months.

**AT8 immunofluorescence**

Three 20µm free-floating mouse brain sagittal sections were washed in PBS with gentle shaking, and sections were blocked with 0.5% BSA/0.3% TX100 in PBS for 1 hour at room temperature. Sections were incubated with biotinylated phospho-tau (Ser202, Thr205) monoclonal AT8 (1:500, Thermo Fisher, #MN1020B) primary antibody in 0.5% BSA/0.3% TX100 in PBS at 4^o^C overnight. On the next day, sections were washed in PBS with gentle shaking, followed by incubation with Alexa Fluor® 594 conjugated streptavidin (1:200, BioLegend, #405240) secondary antibody in 0.5% BSA/0.3% TX100 in PBS for 2 hours at room temperature with gentle shaking. Subsequently, sections were washed in PBS and mounted onto superfrost plus slides (Ted Pella, Cat# 260100) and allowed to air dry at room temperature. Finally, sections were coverslipped with an aqueous mounting medium (Vector laboratory, #H-5501), followed by sealing with nail polish and air drying. Brain tissue sections were scanned on a Nikon Ti-E Confocal Microscope (Nikon Instruments Inc) and five images per mouse in the subiculum were acquired at 40X with NIS element software. The image quantification was performed with the NIH Image J software (Version 1.53) for the AT8 stain-positive area, which was expressed as a percentage of the total analyzed area. All the images were analyzed by two observers blinded to the experimental group.

**References**

1. Ou W, Yang J, Simanauskaite J, Choi M, Castellanos DM, Chang R, Sun J, Jagadeesan N, Parfitt KD, Cribbs DH, Sumbria RK: **Biologic TNF-α inhibitors reduce microgliosis, neuronal loss, and tau phosphorylation in a transgenic mouse model of tauopathy.** *J Neuroinflammation* 2021, **18:**312.
