# Supplementary material for: Modulation of hippocampal protein expression by a brain penetrant biologic TNF-α inhibitor in the 3xTg Alzheimer’s disease mice
Source: J Transl Med. 2024 Mar 18;22:291. doi: 10.1186/s12967-024-05008-x (PMC10946165; doi:10.1186/s12967-024-05008-x)
Supplement: Supplementary file 12 — Additional file 12: Table S2. List of differentially and non-differentially expressed proteins in Tg-TfRMAb-TNFR compared with Tg-Saline 3xTg-AD mice. [file 12967_2024_5008_MOESM12_ESM.docx]

**Table. S2.** **List of differentially and non-differentially expressed proteins in Tg-TfRMAb-TNFR compared with Tg-Saline 3xTg-AD mice.**

|  | **Subiculum** | **CA2 and DG** |
| --- | --- | --- |
| **Proteins** | **Differentially expressed?** | **Differentially expressed?** |
| Aldh1l1 | NS | NS |
| Amyloid-Beta 1-42 | NS | NS |
| Amyloid Precursor Protein | NS | NS |
| ApoA-I | NS | NS |
| APOE | NS | NS |
| ATG12 | NS | NS |
| ATG5 | NS | NS |
| BACE1 | NS | Significant |
| BAG3 | NS | NS |
| Beclin-1 | NS | NS |
| Calbindin | NS | NS |
| CD11b | NS | NS |
| CD163 | Significant | NS |
| CD31 | NS | NS |
| CD39 | NS | NS |
| CD40 | NS | NS |
| CD45 | NS | NS |
| CD68 | NS | NS |
| CD9 | NS | NS |
| CSF1R | NS | NS |
| Ctsd | NS | Significant |
| GFAP | NS | NS |
| GPNMB | NS | NS |
| IBA1 | NS | NS |
| IDE | NS | NS |
| ITGAX | NS | NS |
| Ki-67 | NS | NS |
| LC3B | NS | NS |
| LRRK2 | NS | NS |
| MAP2 | NS | NS |
| Mertk | NS | NS |
| MHC II | NS | NS |
| MSR1 | NS | NS |
| Myelin basic protein | Significant | NS |
| Neprilysin | Significant | NS |
| NeuN | NS | NS |
| Neurofilament light | NS | NS |
| NRGN | NS | Significant |
| Olig2 | Significant | NS |
| P2RX7 | Significant | NS |
| P62 | NS | NS |
| Park5 | NS | NS |
| Park7 | NS | NS |
| Phospho-Alpha-synuclein (S129) | NS | Significant |
| Phospho-Tau (S199) | NS | NS |
| Phospho-Tau (S214) | NS | NS |
| Phospho-Tau (S396) | NS | NS |
| Phospho-Tau (S404) | NS | NS |
| Phospho-Tau (T231) | NS | NS |
| PINK1 | NS | NS |
| PLA2G6 | NS | NS |
| PSEN1 | NS | NS |
| S100B | NS | NS |
| SPP1 | NS | Significant |
| Synaptophysin | NS | NS |
| Tau | NS | NS |
| Tdp-43 | Significant | NS |
| TFEB | NS | NS |
| TMEM119 | NS | NS |
| Tyrosine Hydroxylase | NS | NS |
| Ubiquitin | NS | NS |
| ULK1 | NS | Significant |
| Vimentin | NS | NS |
| VPS35 | NS | NS |

NS: non-significant, significant: p<0.05 based on a two-sample t-test or Mann-Whitney U test.
